# Chromatin module inference on cellular trajectories identifies key transition points and poised epigenetic states in diverse developmental processes

**DOI:** 10.1101/gr.215004.116

**Published:** 2017-07

**Authors:** Sushmita Roy, Rupa Sridharan

**Affiliations:** 1 Department of Biostatistics and Medical Informatics, University of Wisconsin–Madison, Madison, Wisconsin 53715, USA;; 2 Wisconsin Institute for Discovery, Madison, Wisconsin 53715, USA;; 3 Department of Cell and Regenerative Biology, University of Wisconsin–Madison, Madison, Wisconsin 53715, USA

## Abstract

Changes in chromatin state play important roles in cell fate transitions. Current computational approaches to analyze chromatin modifications across multiple cell types do not model how the cell types are related on a lineage or over time*.* To overcome this limitation, we developed a method called Chromatin Module INference on Trees (CMINT), a probabilistic clustering approach to systematically capture chromatin state dynamics across multiple cell types. Compared to existing approaches, CMINT can handle complex lineage topologies, capture higher quality clusters, and reliably detect chromatin transitions between cell types. We applied CMINT to gain novel insights in two complex processes: reprogramming to induced pluripotent stem cells (iPSCs) and hematopoiesis. In reprogramming, chromatin changes could occur without large gene expression changes, different combinations of activating marks were associated with specific reprogramming factors, there was an order of acquisition of chromatin marks at pluripotency loci, and multivalent states (comprising previously undetermined combinations of activating and repressive histone modifications) were enriched for CTCF. In the hematopoietic system, we defined critical decision points in the lineage tree, identified regulatory elements that were enriched in cell-type–specific regions, and found that the underlying chromatin state was achieved by specific erasure of preexisting chromatin marks in the precursor cell or by de novo assembly. Our method provides a systematic approach to model the dynamics of chromatin state to provide novel insights into the relationships among cell types in diverse cell-fate specification processes.

Regulatory networks that control cell-type–specific gene expression patterns are established through a complex interplay between epigenetic modifications and transcription factor binding at regulatory regions of a gene. Transcription factors alone are sufficient to convert differentiated somatic cells to induced pluripotent stem cells (iPSCs) (Takahashi and Yamanaka 2006) albeit at low efficiency. Chemical or genetic modifiers that reduce repressive chromatin levels enhance reprogramming efficiency implicating epigenetic contribution (Onder et al. 2012; Apostolou and Hochedlinger 2013; Papp and Plath 2013; Sridharan et al. 2013). Reciprocally, during development, the chromatin state at specific loci has to become permissive concomitant with appropriate transcription factor levels for cell-type–specific expression to commence. Given the multitude of histone modifications and their combinations, parsing which ones are necessary or sufficient to enable a permissive environment for gene expression is a challenge. Therefore, systematic approaches to study the dynamics of chromatin are essential to understand the underlying regulatory networks that drive transitions during cell fate change.

Several computational approaches, ChromHMM (Ernst and Kellis 2010), jMosaics (Zeng et al. 2013), EpiCSeg (Mammana and Chung 2015), Segway (Hoffman et al. 2012), and GATE (Yu et al. 2013), have been developed to examine multiple chromatin marks in one or more cell types. With the exception of GATE, these approaches focus more on automatically segmenting the genome to identify regulatory elements and less on examining dynamics of chromatin state. Most computational analyses of chromatin marks across multiple cell types have either focused on identifying differential regions between pairs of cell types or time points (Liang and Keles 2012; Shao et al. 2012), single clustering of loci using marks across all cell types (Suvà et al. 2014), or clustering entire epigenomes one mark at a time (Roadmap Epigenomics Consortium et al. 2015). Importantly, existing approaches for multiple cell-type chromatin data do not account for the hierarchical relationships between the cell types.

To enable systematic characterization of chromatin state dynamics across multiple related cell types, we developed Chromatin Module INference on Trees (CMINT). We define a chromatin module to be a set of genomic loci with the same combination of chromatin modifications that likely represent coordinately regulated genes exhibiting similar regulatory states analogous to gene expression modules (Tanay et al. 2004). A novel aspect of our approach is that we model the relationship of different cell types.

We applied CMINT to eight chromatin marks to study chromatin state transitions during reprogramming to iPSCs. Seven of these marks correspond to histone post-translational modifications (PTMs) that we previously identified to be significantly changed during reprogramming using an unbiased mass spectrometry approach (Sridharan et al. 2013). These marks are associated with active transcription (H3K4me3, H3K9ac, H3K14ac, and H3K18ac), repression (H3K9me3 and H3K9me2), and transcription elongation (H3K79me2). We profiled these modifications in the promoters of somatic cells, partial and completely reprogrammed iPSCs, and combined it with published data measuring H3K4me3 and H3K27me3 (Maherali et al. 2007; Sridharan et al. 2009). We also applied CMINT to the hematopoietic lineage with 15 different cell types in which four chromatin marks (H3K27ac, H3K4me1, H3K4me2, and H3K4me3) were measured (Lara-Astiaso et al. 2014).

## Results

### CMINT: Chromatin Module INference on Trees

CMINT is a generative probabilistic graphical model-based approach for multitask clustering (Caruana 1997) that simultaneously identifies chromatin modules in multiple cell types. We define a chromatin module as a set of genomic loci with the same chromatin state specified by the combination of histone PTMs (henceforth called marks). Given multiple chromatin marks from multiple cell types related by a tree, CMINT addresses four questions: (1) in what chromatin states do genomic loci exist; (2) to what extent are chromatin modules shared between cell types at the level of mark combinatorial pattern; (3) how likely are genomic loci to switch modules; and (4) which genomic loci switch chromatin state between cell types, since such loci are likely important for cell state change.

CMINT is motivated by the hierarchical structure of developmental lineages, in which a new cell type arises from a predecessor through several intermediate states. Such relationships are naturally represented by a tree, and computational approaches that can incorporate the tree structure while identifying regulatory modules and networks have been useful in understanding evolutionary (Xie et al. 2011; Roy et al. 2013; Shay et al. 2013) and developmental processes (Jojic et al. 2013). CMINT is based on a previous module inference algorithm that we developed for species lineages (Roy et al. 2013) with two major extensions. Specifically, CMINT handles the complex topology of cell lineages that can progress successively into more than two differentiated states, which can vary depending upon the point in the lineage. In addition, unlike a species phylogeny, in which ancestral states are unobserved, in a cell lineage, ancestral cellular states represent a progenitor cell that can be experimentally profiled and therefore needs to be modeled as observed data.

**Figure 1.**
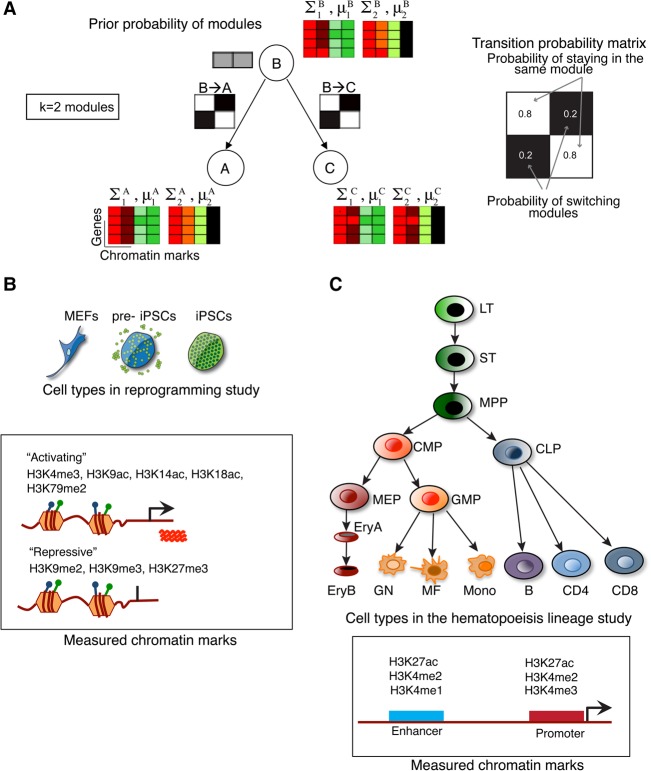
The CMINT approach. (*A*) The generative model of the CMINT approach. The model is made up of two parts: the first part corresponds to a mixture of *P*-dimensional Gaussians, one dimension for each mark. The second part specifies the transition probabilities of genes (black-white matrices) switching modules between a cell type and its predecessor. Each circle on the tree corresponds to a cell type. All cell types other than the root cell type (e.g., the starting differentiated cell type) have a *k* × *k* matrix of conditional probabilities. The starting cell type only has an initial prior probability distribution of module assignments (gray boxes). (*B*) The reprogramming system: (MEFs) mouse embryonic fibroblasts; (pre-iPSCs) partially reprogrammed induced pluripotent stem cells; (iPSCs) induced pluripotent stem cells. *Bottom*: Histone H3 lysine (K) modifications assessed by ChIP-chip analysis, listed according to their association with transcriptional activation or repression when present alone. (*C*) The hematopoietic system: (LT) long-term hematopoietic stem cells; (ST) short-term hematopoietic stem cells; (MPP) multipotent progenitor; (CMP) common myeloid progenitor; (MEP) megakaryocyte erythrocyte precursor; (EryA) immature erythrocytes; (EryB) mature erythrocytes; (GMP) granulocyte monocyte precursor; (GN) granulocyte; (MF) macrophage; (Mono) monocyte; (CLP) common lymphoid progenitor; (B) B lymphocyte; (CD4) CD4 T lymphocyte; (CD8) CD8 T lymphocyte. *Bottom*: Histone modifications profiled in Lara-Astiaso et al. (2014) and their known localization pattern.

The CMINT generative model is made up of two parts (Fig. 1A). The first captures the chromatin modules in a cell type modeled by a mixture of *k* multivariate Gaussian distributions with diagonal covariance (Hastie et al. 2003). The second part captures module transition dynamics of loci between the different cell types with conditional probability distributions, one for each branch in the tree (Fig. 1A, black-white matrices). The conditional probability distribution specifies the probability that a locus is in module *i* in cell type A given its module assignment in the immediate ancestral cell type of A. One of the cell types is designated as the root of the tree at which we have a prior probability of loci to belong to one of the *k* modules. The parameters of the model are the means and variances for each of the *k* Gaussians in each cell type, the prior probability of modules, and the module transition probabilities for each tree branch. We use the Expectation Maximization (EM) algorithm to estimate these parameters (Methods, Supplemental Methods).

CMINT is applicable to chromatin mark profiles measured using microarrays as well as next-generation sequencing technologies. For our reprogramming study (Fig. 1B), we used eight chromatin mark profiles measured using promoter microarrays in three cell types: (1) mouse embryonic fibroblasts (MEFs), the starting differentiated cell type, (2) a stalled intermediate cell state (pre-iPSCs) derived from MEFs (Sridharan et al. 2009), and (3) iPSCs. In this study we generated chromatin immunoprecipitation followed by binding to a promoter microarray (ChIP-chip data) for H3K9ac, H3K14ac, H3K18ac, H3K9me2, H3K9me3, and H3K79me2. To demonstrate the applicability of CMINT both to a more complex lineage structure and for genome-wide sequencing data, we used ChIP-seq data from a comprehensive study of hematopoiesis (Fig. 1C) by Lara-Astiaso et al. (2014) that measured four chromatin marks in 16 different cell types using ChIP-seq.

### CMINT outperforms other approaches to finding modules and transitions

We first compared the quality of CMINT clusters from the reprogramming system (Fig. 1B) to that from two baseline methods (Supplemental Fig. S1A). The MERGE-FIRST approach merges the data matrices per cell type into a single matrix with as many measurements per gene as the total number of marks times the number of cell types and then clusters this merged data matrix (Methods). The per-cell-type clusters are identified by projecting the cluster assignments on to each cell-type–specific data. (Supplemental Fig. S1A, M1). The CLUSTER-FIRST clusters each cell type independently followed by post-processing matching of clusters of one cell type to those from another (Supplemental Fig. S1A, C1). Comparison of the methods based on a silhouette index, which measures the sharpness in boundaries of clusters, shows that the MERGE-FIRST approach produces the lowest quality clusters (Supplemental Fig. S1A). The CLUSTER-FIRST approach, which solely optimizes the cluster quality per cell type, is expected to have the highest silhouette index.

We next evaluated CMINT and the CLUSTER-FIRST approach for their ability to detect chromatin module transitions on simulated data for which the actual transitions were known (Methods). For CLUSTER-FIRST, we used the Hungarian algorithm (Kuhn 2010) to match clusters from one cell type to another. We used Precision, Recall, and *F*-score to compare the cluster transitions to true cluster transitions (Supplemental Fig. S1) and found that CMINT is significantly better (high *F*-scores) compared to CLUSTER-FIRST in detecting transitions (Supplemental Fig. S1B). CLUSTER-FIRST has lower precision (Supplemental Fig. S1C) and comparable recall to CMINT (Supplemental Fig. S1D). This indicates that by using the CMINT approach of jointly clustering the chromatin marks across multiple cell types while exploiting their relatedness, we are able to more reliably detect cluster transitions.

**Figure 2.**
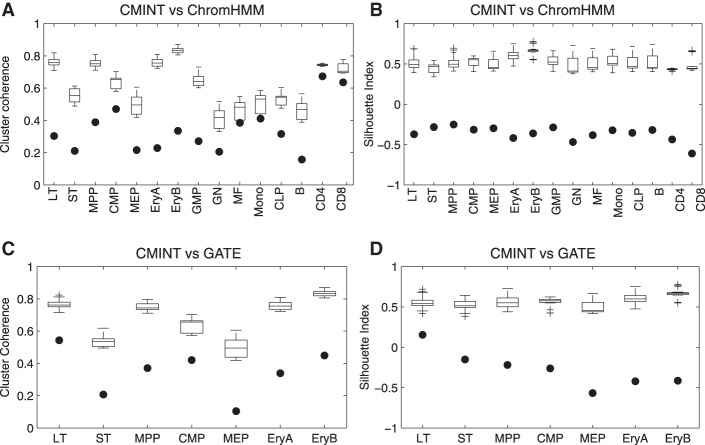
Comparison of CMINT against ChromHMM and GATE on 15 cell types of the hematopoiesis lineage. Cluster coherence (*A*) and silhouette index (*B*) of clusters generated by using ChromHMM and CMINT. The filled circles represent the cluster coherence and silhouette index values for ChromHMM. The box plots represent the values obtained using CMINT on 20 different random initializations. Cluster coherence (*C*) and silhouette index (*D*) of clusters generated by using GATE and CMINT on one branch of hematopoietic tree. The filled circles represent the silhouette index and cluster coherence values for GATE. The box plots represent the values obtained by CMINT for 20 different runs of the algorithm.

We next compared the output of CMINT from the hematopoietic hierarchy (Fig. 1C) to that from two other methods that have been used to examine multiple chromatin marks across cell types—ChromHMM (Ernst and Kellis 2010) and GATE (Yu et al. 2013). By using the entire hematopoietic lineage, we found that the cluster quality of CMINT is better than that of ChromHMM based on cluster coherence (Fig. 2A) and silhouette index (Fig. 2B). The clusters obtained by CMINT were also visually better than ChromHMM (Supplemental Fig. S2). For comparison to GATE, which is suited for time courses, we restricted ourselves to the single longest branch, selecting the erythrocyte lineage, which has six cell types: LT, ST, CMP, MEP, EryA, and EryB. CMINT again performed better in terms of cluster quality using both coherence and silhouette index (Fig. 2C,D) and patterns in the heatmaps (Supplemental Fig. S3). Taken together these results demonstrate the advantage of using CMINT to study chromatin state dynamics on cell lineages.

### CMINT provides novel insights into the chromatin state dynamics during reprogramming

Using CMINT, we first determined the most likely trajectory among two possibilities: a linear chain on which MEFs led to pre-iPSC, which led to the iPSC state, and a branch on which MEFs led to pre-iPSC and iPSC states independently. Based on the model likelihood, we found that the linear chain in both directions, from MEFs to iPSCs and from iPSCs to MEFs, was significantly higher than the branching relationship (*t*-test *P*-value <10^−19^) (Supplemental Fig. S4A). Furthermore, both these linear trajectories are much more likely than other possible linear trajectories, suggesting that the chromatin state in pre-iPSCs is on the trajectory to acquire the iPSC state.

**Figure 3.**
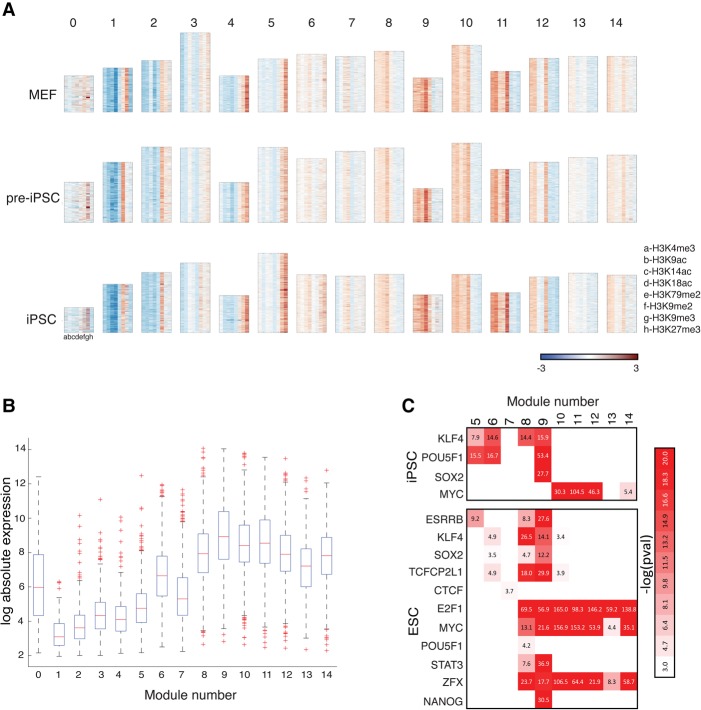
Chromatin modules in the reprogramming cell types identified by CMINT. (*A*) Heatmaps of 15 chromatin modules ordered from 0–14, obtained from CMINT: (*top*) MEFs; (*middle*) pre-iPSC; (*bottom*) iPSC. Each row in each heatmap represents one gene; each column represents one histone modification. (Red) enriched; (blue) depleted as compared to input. Height of each module is roughly proportional to the number of genes. (*B*) Box plots of gene expression of genes in each of the chromatin modules in iPSC. (*C*, *top*) Enrichment of reprogramming factors in the iPSC modules based on ChIP-chip data from iPSCs; (*bottom*) enrichment of pluripotency factors in the iPSC modules based on ChIP-seq data from ESCs.

We used CMINT to identify 15 modules in each cell type (Fig. 3) based on the average of the number of modules that would be selected for each cell type (Methods; Supplemental Fig. S4B). Five modules (1, 2, 3, 4, and 5) were enriched for repressive marks; six modules (8, 9, 10, 11, 12, and 14), were associated with activating marks, and two modules (6 and 7) were associated with both activating and repressive marks (Fig. 3A). Two modules (0 and 13) were not as coherent as other modules but contributed to transitioning genes as discussed below. The activating modules were organized into three distinct patterns: Module 9 was enriched for all the activating marks (with some enrichment for H3K9me3 in iPSCs), whereas module 8 only excluded H3K79me2. Modules 10–14 were specifically depleted for H3K14ac and H3K18ac to varying extents. Modules 6 and 7, which were enriched for both activating and repressive modifications, represent novel “multivalent” modules. We define multivalent modules as those containing multiple activating and repressive marks, although at a resolution of 8 kb they may not occur on the same nucleosome. Gene expression levels (Sridharan et al. 2009) were consistent with chromatin patterns (Fig. 3B; Supplemental Fig. S4C), with modules 8–14 having higher expression than modules 1–5 and the multivalent modules displaying an intermediate expression level (Fig. 3B). For example, module 6 had significantly high expression (*t*-test *P*-value <10^−6^) compared to Modules 1–5, but significantly lower expression compared to modules 8–14 (*t*-test *P*-value <10^−8^). We found a similar trend in MEFs and pre-iPSCs (Supplemental Fig. S4C), suggesting that the combination of modifications in each module and their relationship to expression is a conserved property across multiple cell types.

We determined the biological significance of the modules based on enrichment of transcription factor binding and functional categories using false discovery rate (FDR)-corrected hypergeometric test *P*-value (FDR < 0.05). We used ChIP-chip binding data of the reprogramming factors POU5F1 (also known as OCT4), SOX2, MYC, and KLF4 in iPSC (Sridharan et al. 2009) and pluripotency proteins in embryonic stem cells (ESCs) (Fig. 3C; Chen et al. 2008). Module 9, which contained all the activating marks, was enriched for binding of POU5F1, KLF4 or SOX2, and NANOG. Intriguingly, modules 10, 11, and 12, which were relatively depleted for H3K14ac and H3K18ac, were enriched for MYC binding in iPSCs and not enriched for any of the other factors (Fig. 3C). The combination of MYC (includes both MYC and MYCN), E2F1, and ZFX were again enriched in the modules that lacked H3K14ac and H3K18ac in ESCs (Fig. 3C). The multivalent module 6, which is depleted for H3K9ac, H3K79me2, and H3K9me2, is enriched for POU5F1, SOX2, KLF4, ESRRB, and TCFCP2L1 binding, but not for MYC. Intriguingly, the multivalent module 7, characterized by H3K14ac, H3K18ac, and H3K9me2 is enriched only for the 3D chromatin organizing protein CTCF. Taken together, these results suggest that the reprogramming factors likely recruit different chromatin modifying complexes to achieve high expression of genes in iPSCs, and some multivalent states may not control gene expression but instead organize the genome into territories as evidenced by the enrichment of CTCF.

Enrichment analysis of Gene ontology processes (FDR < 0.05) (Supplemental Fig. S4D; Ashburner et al. 2000) revealed that the repressive modules 1–4, which were enriched for H3K9me2 and H3K9me3, included sensory transduction families of olfactory and taste receptors, protocadherins, and cytokine genes, whereas module 5 included transcription factors related to morphogenesis. Modules 0 and 13, which had higher expression than modules 1–4 were enriched for cation binding (0) and mitochondria related processes (13). Although the modules 10–14 exhibited similar binding profiles (Fig. 3), they were functionally separable into distinct metabolic processes including protein catabolism (10), RNA translation (11), vesicle transport (12), and kinase activity (14). These results further demonstrate that the CMINT approach is adept at identifying biologically relevant clusters.

### Cell-type–specific transitions suggest that the bottleneck to reaching the pluripotent state is in gene activation

**Figure 4.**
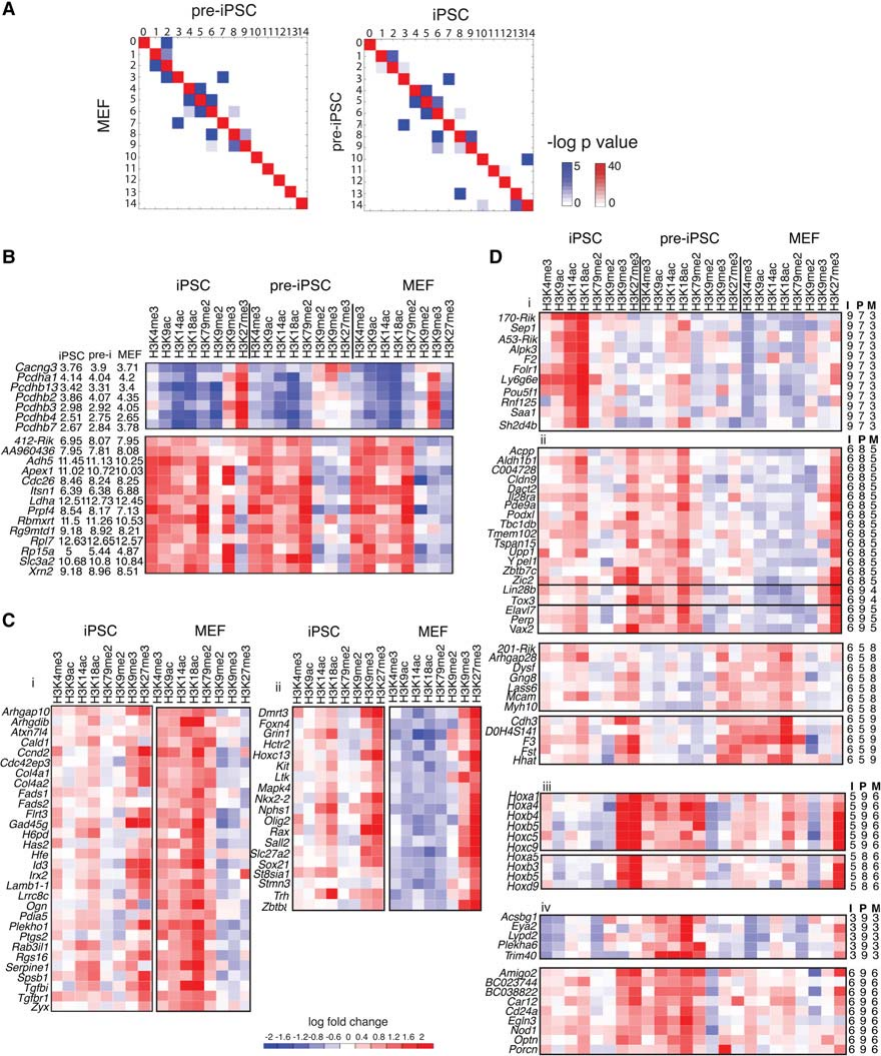
Chromatin module transitions during reprogramming. (*A*) Plot of similarity of module membership of genes that change between MEFs and pre-iPSCs (*left*), and pre-iPSCs and iPSCs (*right*). Two different colors are used: (red) denotes similarity for modules with the same pattern (diagonal entries); (blue) denotes similarity for modules with different patterns (off-diagonal entries). The more red or blue an entry, the more similar are the matrices. The intensity of red (blue) corresponds to the significance of overlap of regions (genes) between two cell types and is the mean of the negative log of two hypergeometric test *P*-values. One *P*-value uses regions from one cell type as the background, and another *P*-value uses the regions from the second cell type as the background. (*B*) Example sets of genes that do not change greatly in expression but change in module membership. (*Left*) Gene names and log gene expression in iPSC, pre-iPSC (pre-i) and MEF. (*Right*) Heat map of enrichment of all histone modifications in iPSC, pre-iPSC and MEF compared to input. (Red) enriched; (blue) depleted. (*412-Rik*) *4121402D02Rik*. (*C*) *Left* and *right* panels represent different gene sets, each exhibiting a different type of chromatin transition. (*Left*) Example set of genes that gain multivalency in iPSCs from an active state in MEF. (*Right*) Example set of genes that gain multivalency in iPSCs from a repressed state in MEF. Gene names are provided on the *left*. The heat maps show the enrichment of histone marks compared to input in each cell type. (*D*) Chromatin module dynamics of genes identified using rules encoding patterns. The *right* panels provide module membership of genes in (I) iPSC, (P) pre-iPSC, and (M) MEF. Gene names are provided on the *left*, histone modifications in red–blue heatmaps: (red) enriched; (blue) depleted. (i) Example of genes that transition through a multivalent state (module 7) in pre-iPSC to an active state in iPSCs from a repressive state in MEF. (ii) Examples of gene sets that transition to a multivalent state in iPSC (module 6) through activating modules (module 8, 9) in pre-iPSCs (*top*) or a repressed modules (module 5) in pre-iPSCs (*bottom*). (iii) Example sets of genes that acquire transient active modifications in pre-iPSC. (iv) Example sets of genes that display an aberrant activated state in pre-iPSC that is not recapitulated in the starting MEF or endpoint iPSC cell types: (*170-Rik*) *1700061G19Rik*; *(201-Rik*) *2010002N04Rik*.

Although similar modules are observed in all three cell types, the genes exhibiting a specific pattern may not be the same between cell types. Hence, we examined the similarity of the genes in modules between pairs of cell types (Fig. 4A) by creating a module similarity matrix using the average of the negative logarithm of two hypergeometric test *P*-values (one for each cell type's regions as the background). The red diagonal in each pairwise comparison indicates a high similarity in the genes in modules with the same pattern across the cell types (Fig. 4A). We identified the module pairs between which genes tended to change patterns in the different cell types by inspecting significant off-diagonal entries (hypergeometric test *P*-value <0.05) (Fig. 4A, blue entries). The significance of the transitions depended both on the modules and cell types being compared and ranged from 0.02 to 10^−43^ (Fig. 4A, blue intensity). Strikingly, genes tended to switch between repressive (1–5) or multivalent modules (6 and 7) in the MEF-pre-iPSC transition more than between active modules (9–14). In contrast, the transitions between pre-iPSCs and iPSCs occur between repressive, activating, and multivalent modules. This switching pattern suggests that genes in pre-iPSC retain the MEF activation pattern, and the bottleneck to reaching the iPSC state is likely in the activation of genes. From the multivalent module 6, genes could transition to both repressive (modules 4 and 5) and activating (modules 8 and 9) states. Genes in multivalent module 7 only transition to module 3 by losing active marks, irrespective of cell type. Transition of genes in activating modules tended to occur within modules 8 and 9 (enriched for POU5F1, SOX2, KLF4) or between modules 10–14 (enriched for MYC). From module 0, genes tended to transition to module 2 and from module 13 to module 8.

Taken together from this global perspective, we find that (1) significant numbers of genes do not transition between activating to repressive patterns directly; (2) the repressive modules in MEFs have transitioned to an activated state in pre-iPSC, suggesting that gene activation rather than repression could be the bottleneck in transitioning to iPSCs from the pre-iPSCs; and (3) not all multivalent modules can resolve into both activating and repressive patterns.

### Changes in chromatin state can occur without large changes in gene expression

We next investigated whether there were genes that exhibited low expression changes or expression-independent transitions in chromatin state, because such changes could indicate a poised state responsive to environmental cues. We grouped genes into high (log[Expression] > 9.2), medium (5.92 < log[Expression] < 9.2), and low expression levels (log[Expression] < 5.92) by fitting a Gaussian mixture model to the logarithm of the absolute expression levels measured in these cell types previously (Sridharan et al. 2009). Focusing on genes with both mRNA and chromatin measurements, we found a total of 6310 genes that changed their chromatin state between any two cell types. Of these, about a third (2317) changed their expression state, suggesting that the majority of the genes change their chromatin state without very large changes in expression. However, most such transitions of genes occurred between modules of similar expression outputs. For example, the protocadherin (*Pcdh*) genes involved in cell adhesion switched from module 1 (repressive with H3K9me2 and H3K9me3) in MEFs to module 2 (repressive with H3K27me3) in iPSCs (Fig. 4B, top). Similarly, a shift from activating module 12 in MEFs to another activating module 10 in pre-iPSCs and iPSCs does not change the expression levels drastically (Fig. 4B, bottom).

### Transitions at individual loci identify multiple routes to multivalency and bottlenecks to iPSCs

Using the transitions between cell types, we asked whether the multivalent state 6 in iPSCs arises primarily because of preexisting repressive marks in MEFs that then acquire activating marks or vice versa (Fig. 4C). We found examples of both kinds of transitioning genes (761), with the majority arising from activating modules in MEFs (477 genes). In contrast, a significant fraction (67%) of the genes in the multivalent module 7 of iPSCs arose solely from module 3 of MEFs. Genes that transitioned from having repressive modifications in MEFs (Fig. 4C,i) to becoming multivalent in iPSCs were enriched for NANOG, ESRRB, and SOX2 binding (FDR < 0.05), suggesting that these transcription factors were required to add on to the preexisting activating modifications.

Since our trajectory analysis indicated that the pre-iPSC state was intermediate to that of MEFs and iPSCs (Supplemental Fig. S4A), we examined transitions encountered in the pre-iPSC state (Fig. 4; Supplemental Material). This allowed us to examine the pre-iPSC state of genes that have opposing chromatin module memberships in MEFs and iPSCs. We coded these “rules” using module membership in the three cell types: iPSC–pre-iPSC–MEF. For example, the transition 5-6-8 meant that the gene was in a repressive module 5 in iPSC, in the multivalent module 6 in pre-iPSC, and activating module 8 in MEF. We found examples of such genes to be in multivalent (9-7-3) (Fig. 4D,i) and activating states (9-8-5) in pre-iPSCs (Supplemental Material).

We then examined the genes that became multivalent in iPSCs. Interestingly, if this state was reached from an active state in MEF, then in pre-iPSC, the genes were first repressed completely before acquiring the active modification in iPSC. This group (6-5-8 and 6-5-9) (Fig. 4D,ii, bottom) was enriched for TCFCP2L1 binding in iPSCs (FDR < 0.05). Conversely, genes acquired a transient active state in pre-iPSCs from a repressive state in MEFs before reaching multivalency in iPSCs including patterns 6-8-5, 6-9-5, 6-9-4, 7-9-2, 7-9-3 (Fig. 4D,ii, top, examples). There are also genes that are in a multivalent state in MEFs and pass through an activated state in pre-iPSCs before being repressed (5-8-6, 5-9-6). This group contains several patterning genes of the *Hox* clusters (Fig. 4D,iii). Finally, genes could display a different chromatin state in pre-iPSC when the MEF and iPSC states were equivalent including patterns 5-8-5, 5-9-5, 3-9-3, 5-6-5, 6-5-6, and 6-9-6 (Fig. 4D,iv, examples).

### Chromatin states in hematopoietic cell lineage are maintained despite extensive dissimilarity of individual regions exhibiting these states

The previous analysis demonstrated the power of using CMINT in cellular reprogramming but focused on promoter arrays and three cell types. To examine chromatin state dynamics in a more genome-wide setting in a complex hierarchy of cell types, we applied CMINT to 15 cell types of the hematopoiesis lineage using the data set from Lara-Astiaso et al. (2014) measuring four marks, H3K4me1, H3K4me2, H3K27ac (enhancer enriched), and H3K4me3 (promoter enriched) (Fig. 1C). We applied CMINT to 1,189,496 regions of 2000-bp length that were measured in at least one cell type for at least one mark and learned 16 modules in each cell type (Supplemental Fig. S5A). Application of CMINT to these roughly million regions revealed modules with different chromatin signatures. Despite there being overall similarity in the pattern exhibited in different cell types, we observed extensive off-diagonal transitions assessed using multiple measures (Supplemental Fig. S6, *F*-score; Supplemental Fig. S7, significance of overlap). This is an interesting contrast to the reprogramming study (Fig. 4A), in which the majority of the modules had similar genes and is likely due to both the more complex hierarchy and the fact that we examined the entire genome as opposed to genic regions. Among all cell type comparisons, module 15, which was highly enriched for all four marks, had the most similar module membership, followed by modules 14 and 8 (Supplemental Figs. S5A, S6, S7). Closely related cell types (e.g., EryA and EryB) had more similar modules than distant cell types (Supplemental Fig. S6). To functionally interpret these modules, we performed region-level enrichment analysis by mapping known *cis*-regulatory elements from the ORegAnno database (Lesurf et al. 2016) to these regions (Supplemental Figs. S5B, S8; Methods). To examine common regulatory programs across the multiple cell types in the hematopoiesis lineage, we selected regulators that were enriched in the majority of the cell types (at least 12 of 15 cell types). Enrichment analysis of the clusters using these mapped elements recapitulated several known important regulators of the hematopoietic lineage. Several transcription factor elements, including BHLEH40, BCL6, GFI1B, MEIS1, and STAT6 were enriched in all the cell types in module 15, which contains all four marks (Supplemental Fig. 5B). Interestingly, GATA1, a transcription factor that is essential for erythropoiesis, was enriched in module 14 that is relatively depleted in H3K4me3 (Supplemental Fig. 8), a promoter-specific mark, but not in module 15 in the MEP, EryA, and EryB cell types, which had all four marks. This suggests that GATA1 may be more important at the enhancer-like locations specified by H3K27ac and H3K4me1 than at promoter proximal locations in these erythroid lineages. In addition to these activators, we also found a strong enrichment for components of the polycomb response element 2 (PRC2) complex, including JARID2, SUZ12, and EZH2 in all the cell types in module 2, which lacks only H3K27ac (Supplemental Fig. 5B). Since the PRC2 complex catalyzes H3K27me3, this observation would imply that these regions are likely to contain this repressive mark in addition to the H3K4me3 mark, making them bivalent in nature.

**Figure 5.**
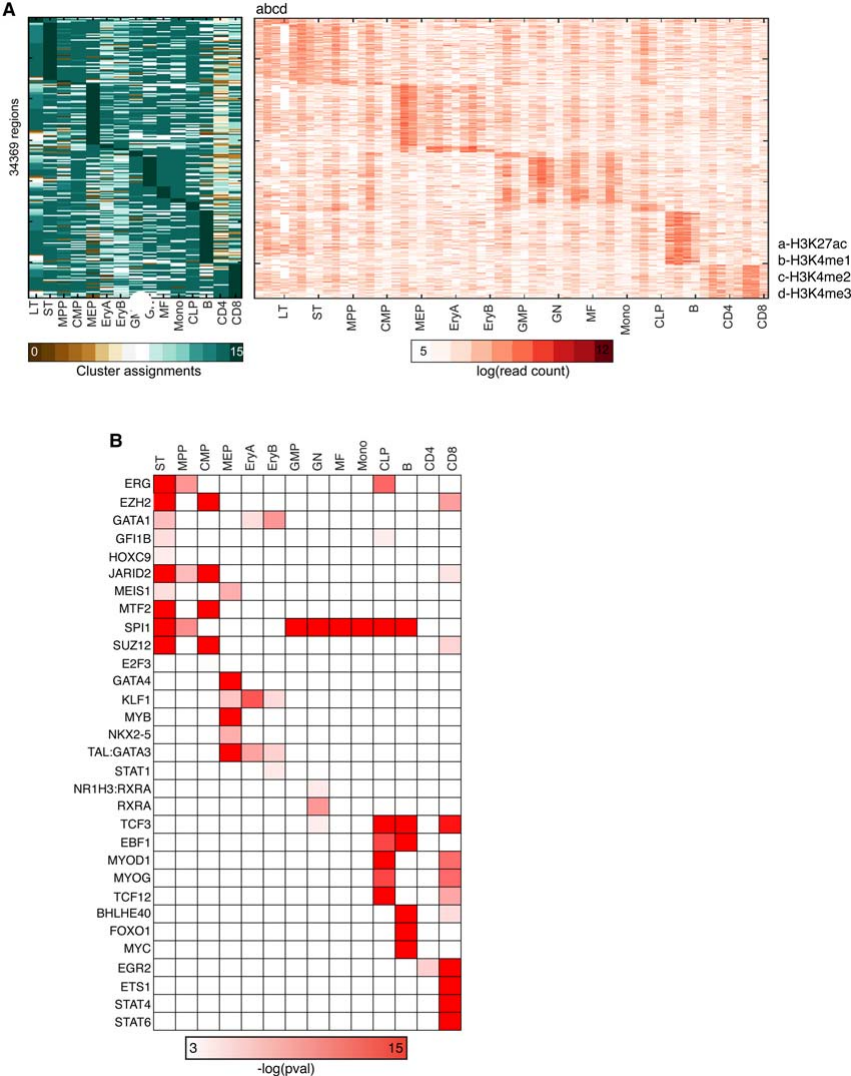
Cell-type–specific regions and decision points identified by CMINT in the hematopoietic hierarchy. (*A*, *left*) Regions that uniquely belong to module 15 in Supplemental Figure S5 and their cluster assignments in other cell types; (*right*) enrichment of each histone modification in the regions that uniquely belong to Module 15 in Supplemental Figure S5. (*B*) ORegAnno *cis*-regulatory element enrichment for factors (*left*) enriched in regions uniquely assigned to module 15 in each of the cell types indicated on *top*.

Given that module 15 had the largest number of common *cis*-regulatory site enrichments, we next sought to determine the factors that may be important at the transition points of the hematopoietic lineage by identifying regions unique to module 15 in each cell type. The number of unique regions in module 15 for each cell type varied significantly, ranging from 113 to 7,536 regions, suggesting that large transitions in module 15 membership occurred only at certain points in the lineage (Fig. 5A). The largest number of transitions occurred at the ST, MEP, GMP-GN-MF-Mono, and B-CD4-CD8 cell types, suggesting that these could be control points (Fig. 5A). *Cis*-regulatory element analysis (Fig. 5B) revealed that the entire PRC2 complex and the transcription factor ERG are both enriched in the ST cluster. Since the PRC2 complex methylates H3K27, during this transition, several regions are likely to be in a bivalent state with H3K27me3 (a repressive mark), present along with H3K4me3 (an activating mark). In the MEP transition, several important regulators of erythropoiesis, including TAL-GATA1 and KLF1, are enriched. Similarly, the unique regions in B cells are enriched for BHLHE40 and FOXO1 motifs, and T cells are enriched for STAT4 and STAT6 (Fig. 5B). Comparison of these enrichments identified in cell-type–specific regions to those for entire modules can postulate candidate cell-type–specific regulators. Although some factors such as STAT6 are enriched in every module 15 (Supplemental Fig. S5B), they are enriched in the unique regions of module 15 only in CD8 T cells, a cell type in which STAT6 is likely critical. This would suggest that the other factors enriched in module 15 of CD8 T cells, such as GFI1B (Supplemental Fig. S5B), are not specific to CD8 T cells as they are absent from regions that are uniquely enriched in module 15 (Fig. 5B). Taken together, these results imply that there are distinct transition points in chromatin state in the hematopoietic hierarchy and specific transcription factors may be involved in setting up the chromatin state at these points.

### CMINT output identifies critical chromatin-level decision points in hematopoiesis

**Figure 6.**
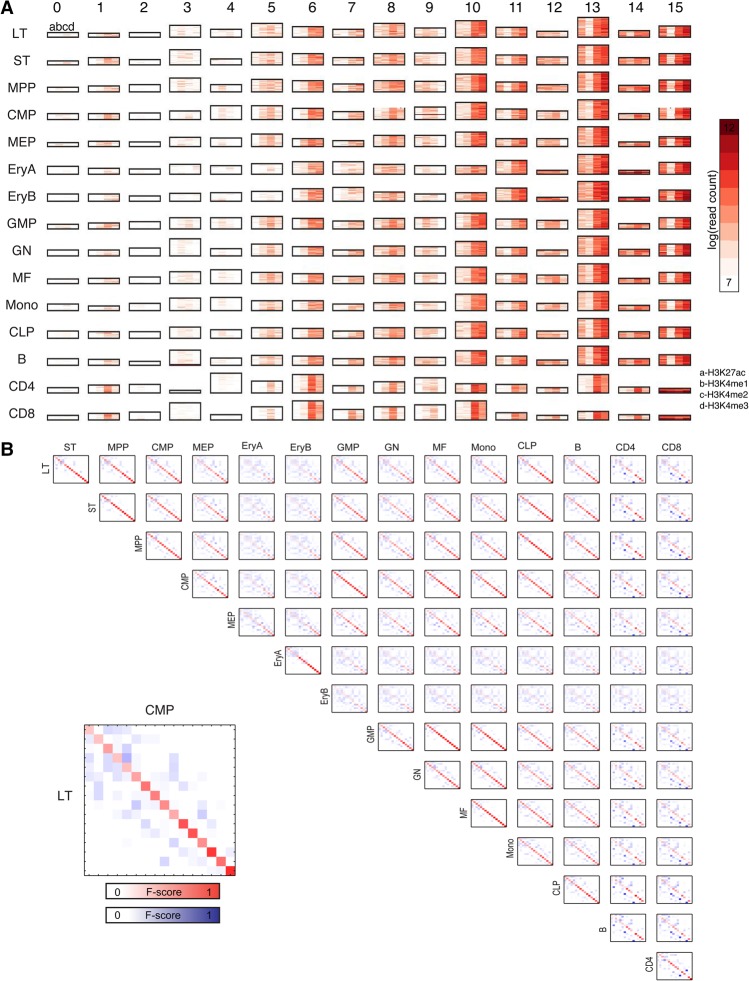
CMINT modules identified on the hematopoiesis cell lineage when applied to a subset of regions containing measurements for all histone modifications. (*A*) Heatmaps of 16 chromatin modules numbered from 0 to 15, obtained from CMINT restricted to 2000-bp regions with a non-zero value for each of the four histone modifications. Each row in each heatmap represents one region; each column represents one histone modification: (red) enriched; (white) depleted. The height of each module is roughly proportional to the number of regions within it. (*B*) Plot of similarity of module membership of regions, in which similarity was measured based on *F*-score, between each pair of cell types. Two different scales are used: (red) similarity for modules with similar patterns (diagonal entries); (blue) similarity for modules with different patterns (off-diagonal entries). The more red or blue an entry, the more similar are the matrices.

Because several modules in the previous analysis had a complete absence of one or more marks, we next applied CMINT to a smaller set of 28,418 regions with non-zero values for all marks in all cell types (Fig. 6A). Among modules learned from these regions, some had lower presence of all the marks (0–4), whereas others were associated with a subset of marks. For example, modules 13, 14, and 15 were associated with H3K27ac, H3K4me2, and H3K4me3, but lower H3K4me1. Module similarity matrix based on both an *F*-score (Fig. 6B) and statistical significance of overlap (Supplemental Fig. S9) recapitulated the known lineage hierarchy (Fig. 6B). In particular, EryA and EryB; GMP, MF and Mono; and CD4 and CD8 were more similar to each other, respectively, than other cell types (Fig. 6B). The most terminally differentiated cell types EryA, EryB, CD4, and CD8 were most dissimilar from other cell types (Fig. 6B).

To further examine the ability of CMINT to discriminate between different lineage structures, we considered eight alternative tree topologies that differed from the original lineage structure at the origin of specific subtrees (Supplemental Fig. S10). Among the different tree topologies, the original tree ranked second, but the likelihood of the first two trees were not significantly different (*t*-test *P*-value <0.05). Three trees that had significantly lower likelihood compared to the original tree differed in the origin of GMP (CLP, MEP, or MPP), suggesting that the transition to GMP requires specific transcription programs to be triggered at the CMP state that is removed later in the lineage (MEP) or has not occurred yet earlier in the lineage (MPP or CLP). These results further highlight the ability of CMINT to select among alternative tree topologies and suggest potential refinements to the tree based on the data likelihood from chromatin marks.

**Figure 7.**
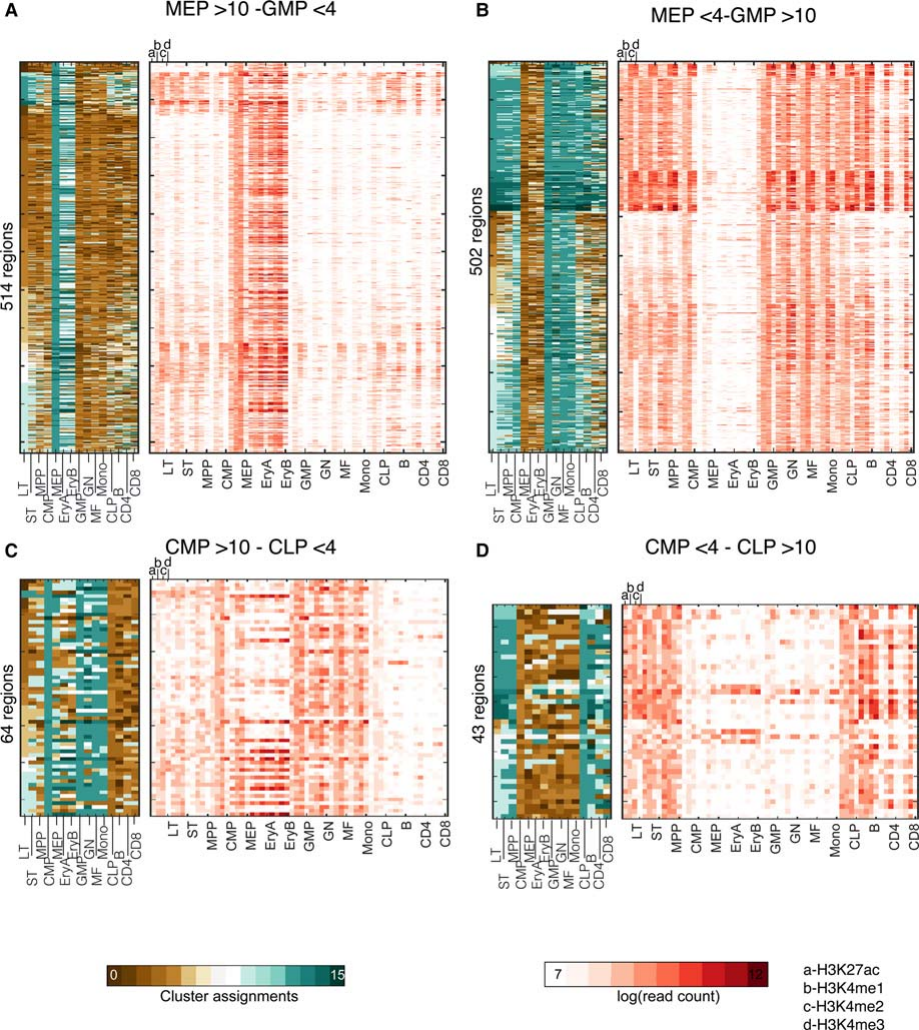
Rule-based analysis of hematopoiesis CMINT modules identifies regions associated with chromatin state transitions at different lineage points. (*A*, *left*) Regions that belong to modules enriched for marks (numbered greater than 10) in MEP and depleted for marks (less than 4) in GMP and their module assignments in other cell types. (*Right*) Histone modification level in the regions that obey the MEP > 10 and GMP < 4 module membership rule. (*B*) Similar to *A*, but for regions that obey the GMP > 10 and MEP < 4 rule. (*C*) As in *A*, but for regions that obey the CMP > 10 and CLP < 4 rule. (*D*) As in *A*, but for regions that obey the opposite rule, CLP > 10 and CMP < 4.

We used CMINT's inferred module assignments to find genomic regions that transitioned between distinct modules at different points in the lineage (Fig. 7). As a proof of principle, we focused on transitions at two points in the tree: CMP versus CLP and the erythroid (MEP) versus macrophage (GMP) lineages (Supplemental Material). We looked for regions that were highly enriched for the modifications in MEP module (>10) but not in GMP (<4) (Fig. 7A), and those that followed the reciprocal rule (Fig. 7B). Both transitions had approximately 500 genes that followed the rule but displayed interesting patterns in the other cell types. For the regions that were in modules >10 in MEP, the chromatin state is established in the MEP cell type and gets much further enhanced in the downstream EryA and EryB lineages (Fig. 7A). These regions are enriched in GATA1, KLF1, MYB, and TAL1 motifs (FDR < 0.05). In striking contrast, the regions that are found in modules greater than 10 in GMP specifically lack mark enrichment in the MEP lineage (Fig. 7B). For these regions, there is some enrichment of the marks in precursor LT and CMP cells, with retention in the downstream cell types, but absence in the completely differentiated lymphoid lineages. These regions were enriched for motifs of the ETS1, SPI1, and ERG factors. We performed a similar analysis for an earlier stage of the lineage, at the CMP versus CLP transition and applied the same rule (Fig. 7C), which yielded fewer regions (50–150) than the MEP/GMP transition. The chromatin state is set up in the CMP cell type and is then retained in the downstream MEP and GMP lineages (Fig. 7C). In the reciprocal transition, there seems to be a specific loss of modifications in the CMP lineages from the earlier progenitors of LT, ST, and MPP and retention in the CLP lineages (Fig. 7D). Taken together these transition patterns suggest that the chromatin state is retained in only one or the other cell type, implicating a further selective process for adding or removing chromatin marks or transcription factor binding.

## Discussion

Dynamics of chromatin state in complex cell-fate specification problems is poorly understood. A significant advantage of our new computational method, CMINT, for studying chromatin state is the ability to model the hierarchical relatedness among cell types while simultaneously defining matched chromatin states across multiple cell types. Therefore, from the output of CMINT, it is simple to query whether particular transitions occur by constructing specific rules that capture patterns of module transitions between the cell types, and to identify genes that exhibit such patterns, as found for reprogramming (Fig. 4) and hematopoiesis (Fig. 7).

In reprogramming, by examining modules for enrichment of transcription factor binding, we identified specific divisions of labor. MYC binding is enriched in clusters that are depleted for H3K14ac and H3K18ac, but that contain H3K9ac and H3K79me2. MYC is known to enhance the transcription elongation rate (Rahl et al. 2010), leading to the interesting insight that perhaps rapid elongation is incompatible with high levels of H3K14ac and H3K18ac. Multivalent module 7, which has the opposite epiphenotype of increased H3K14ac and H3K18ac, is enriched for CTCF, suggesting roles for these modifications in setting up chromosome territories.

A current unanswered question in gene regulation is the necessity or sufficiency of multiple activating modifications for transcription. In the MEF state, *Pou5f1,* which encodes a key pluripotency transcription factor, is repressed by H3K27me3 and H3K9me3 and does not have any activating marks. In pre-iPSCs, where *Pou5f1* is not expressed, all the repressive marks have been erased and the activating H3K14ac and H3K18ac are enriched; in iPSCs, all the activating marks are present. Thus erasure of repressive marks and the gain of H3K14ac and H3K18ac are insufficient to activate the gene. From this starting point, we can use the CRISPR-Cas9 system to interrogate whether these two modifications set up a platform for recruiting the next set of chromatin remodelers to activate *Pou5f1* and result in an iPSC state.

Enrichment of transcription factors among transitioning genes also enabled us to predict regulators that could accelerate the reprogramming process. We have recently found that pre-iPSC can be converted to iPSC by adding vitamin C and a MEK and GSK inhibitor (2i) (Tran et al. 2015), which activate NANOG and TCFCP2L1 expression, respectively. NANOG is enriched in a gene set that acquires an activating state in iPSCs from a multivalent state in pre-iPSCs, suggesting that the expression of *Nanog* may make the chromatin state more conducive for high expression. TCFCP2L1, a component of LIF signaling (Martello et al. 2013; Ye et al. 2013), is enriched in genes that become multivalent in iPSCs from a repressed state in pre-iPSCs, suggesting a function in poising of gene expression.

In the hematopoiesis system, CMINT analysis revealed a remarkable plasticity in chromatin state. CMINT's outputs were useful to identify both regions that were cell-type specific and additionally identify important transition points in the hierarchy. We found that the number of regions that transition depends upon the point in the tree, with more differentiated terminal cell types containing more regions that were distinct between alternative cell types derived from the same progenitor. Some transcription factors (MEIS1 and ERG) that we found to be enriched at the important decision points were also found in the original Lara-Astiaso et al. (2014) work using ATAC-seq and motif enrichment corroborating our conclusions. Interestingly, we found additional elements of the PRC2 complex that repress gene expression at specific points in the lineage tree.

Our current study focused on relatively large genomic regions, aggregating signals either 8 kb for the reprogramming study or 2 kb for the hematopoietic lineage study. However, as more deeply sequenced data become available, a future extension of our work is to scale to higher resolution data to capture more fine-grained interactions among chromatin marks. As more epigenomes are measured for multiple cell types, time points, and conditions (Roadmap Epigenomics Consortium et al. 2015), approaches like CMINT will become increasingly useful to examine the chromatin state dynamics and identify important epigenetic transitions changing global cellular states.

## Methods

### ChIP-chip experiments

ChIP-chip experiments were performed exactly as described in Sridharan et al. (2009). Data for H3K4me3 and H3K27me3 for the iPSC line has been previously published in Sridharan et al. (2009). Antibodies used were H3K4me3 (Abcam ab8580), H3K27me3 (Millipore 07-449); H3K9ac, H3K14ac, and H3K18ac were kind gifts of Prof. Michael Grunstein at UCLA; H3K9me2 (Abcam ab1220), H3K9me3 (Abcam ab8898), H3K79me2 (Activ motif-39143). Elutes were amplified using the Sigma WGA kit and applied to Agilent mouse promoter array (G4490) according to the manufacturer's instructions. Average probe signals were initially extracted in a 500-bp window-stepwise manner as described previously (Maherali et al. 2007) and then averaged across the entire 8000-bp region and used as input for the CMINT algorithm.

### Overview of the CMINT algorithm

CMINT uses a generative probabilistic model to jointly learn clusters of genomic loci exhibiting similar chromatin mark combinations in each cell type. The input to CMINT is genome-wide chromatin mark measurements of *m* marks in each of *n* cell types and a tree relating the cell types. There are two components to the model: a mixture of *k* multivariate Gaussian distributions, each Gaussian modeling one of *k* chromatin modules in each cell type, and a set of transition probabilities to model the relationship in chromatin state between a parent and a child cell type. The number of dimensions of the Gaussian equals the number of marks. The parameters of the model are the mean and covariance of the *k · n* Gaussians, the prior probability of modules at the starting cell type and the *k* × *k* transition probability matrices for each cell type with a parent. The parameter estimation uses the expectation maximization algorithm (Dempster et al. 1977). Briefly, the expectation step (E) step computes the probability of a gene's chromatin profile in cell type *c* to be generated in the *k*^th^ Gaussian. The maximization step (M) estimates the mean and variance based on the probabilities of chromatin profiles to be generated by a particular Gaussian, and the transition probabilities using the joint probability of pairs of module assignments for each parent–child cell-type pair. CMINT can also be used for selecting among different tree topologies using the greatest data likelihood. Additional mathematical details of the model are given in Supplemental Methods.

### Application of CMINT on mouse reprogramming data

We applied CMINT to eight chromatin mark profiles, and each mark's value was averaged across an 8000-bp region associated with a gene promoter. For the array data, clustering the probe-level data did not reveal additional patterns that were not already captured by the aggregated signal. To determine the number of modules, *k*, we used MDL penalized test data likelihood with fivefold cross-validation to determine the best number of modules for each cell type by varying *k* from 3 to 25 in increments of 2 (Supplemental Fig. S11). We selected *k* = 15 as the best because it represented the average of the number of clusters. In addition, we also examined CMINT modules for *k* = 20, 25, and 30 and did not find any increase in the number of patterns detected.

We used CMINT's data likelihood to examine multiple possible topologies that could relate the MEF, iPSC, and pre-iPSC cell types. In one, we had a branching topology in which MEF led to pre-iPSC and iPSC as two independent branches. In the remaining, we examined multiple linear trajectories in which each cell type is treated as the starting cell type. We computed the average likelihood of the CMINT models learned in each setting from 40 runs and used the topology with the data likelihood that was significantly higher than other topologies. The linear topology with MEF as the starting cell type to iPSC as the ending cell type, or the exact reverse, had a significantly greater likelihood. We therefore used the linear topology MEF, pre-IPSC, and iPSC topology for our downstream analysis because this also reflects the direction of the reprogramming process.

To interpret the modules and gene sets with module transitions, we assessed enrichment of Gene Ontology processes (Ashburner et al. 2000), curated gene sets from the MSigDB database (Liberzon et al. 2011), and ChIP-seq peaks of known pluripotency factors (Chen et al. 2008). We used an FDR < 0.05 calculated using the Benjamini-Hochberg procedure on hypergeometric test *P*-values to call a module or gene set enriched in a curated set (for details, see Supplemental Methods).

### Application of CMINT to Hematopoiesis cell lineage data

We obtained raw fastq files for four chromatin marks, H3K4me1, H3K4me2m, H3K4me3, and H3K27ac generated by Lara-Astiaso et al. (2014). Reads were aligned to the mm9 mouse genome assembly using Bowtie 2 with default options (Langmead et al. 2009) and filtered using SAMtools with –q3 option for read quality (Li et al. 2009). The counts were aggregated in 2000-bp regions based on Lara-Astiaso et al. (2014), and the data were normalized for sequencing depth. We excluded the NK cell type due to low sequencing depth. Replicates, where available, were collapsed by taking the median. CMINT was applied on a set of 1,189,496 regions that had one chromatin mark in at least one cell type and on a set of 28,418 regions that had a non-zero read count for all cell types and marks. We applied CMINT with 16 modules after log transforming the normalized data. To interpret the clusters, we calculated enrichments of ORegAnno sequence elements in each module (FDR < 0.05) by mapping module regions to sequence elements (Supplemental Methods).

### Software availability

The CMINT software can be downloaded from the CMINT Supplemental Website (http://pages.discovery.wisc.edu/~sroy/CMINT) and also on Bitbucket (https://bitbucket.org/roygroup/cmint), where future releases of the software will be made available.

## Data access

The CMINT code, associated scripts, input data sets, and results from this study are available on the CMINT website (http://pages.discovery.wisc.edu/~sroy/CMINT) as well as Supplemental Material. Processed data sets are available at the CMINT Supplemental Website. Raw data sets have been submitted to NCBI Gene Expression Omnibus (GEO; https://www.ncbi.nlm.nih.gov/geo/) under accession number GSE97222.

## Supplementary Material

Supplemental Material
