# Supplementary material for: Chromatin module inference on cellular trajectories identifies key transition points and poised epigenetic states in diverse developmental processes
Source: Genome Res. 2017 Jul;27(7):1250–62. doi: 10.1101/gr.215004.116 (PMC5495076; doi:10.1101/gr.215004.116)
Supplement: Supplemental Material [file supp_27_7_1250__index.html]

Chromatin module inference on cellular trajectories identifies key transition points and poised epigenetic states in diverse developmental processes — Supplemental Material 

# Chromatin module inference on cellular trajectories identifies key transition points and poised epigenetic states in diverse developmental processes

## Supplemental Material

- Supplemental\_Fig\_S1.pdf
- Supplemental\_Fig\_S2.pdf
- Supplemental\_Fig\_S3.pdf
- Supplemental\_Fig\_S4.pdf
- Supplemental\_Fig\_S5.pdf
- Supplemental\_Fig\_S6.pdf
- Supplemental\_Fig\_S7.pdf
- Supplemental\_Fig\_S8.pdf
- Supplemental\_Fig\_S9.pdf
- Supplemental\_Fig\_S10.pdf
- Supplemental\_Fig\_S11.pdf
- Supplemental\_Materials.zip
- Supplemental\_Methods.pdf
