# Supplementary material for: Chromatin module inference on cellular trajectories identifies key transition points and poised epigenetic states in diverse developmental processes
Source: Genome Res. 2017 Jul;27(7):1250–62. doi: 10.1101/gr.215004.116 (PMC5495076; doi:10.1101/gr.215004.116)
Supplement: Supplemental Material [file supp_gr.215004.116_Supplemental_Fig_S1.pdf]

Supp Fig S1: Computational validation of CMINT on simulated and real datasets. **A.** Silhouette index for modules inferred by applying CMINT and two other baseline methods on the three-cell type reprogramming data. The baseline methods are: MERGE-FIRST (M1), which merges marks by concatenating gene chromatin mark measurements from all cell types and infer clusters by applying a Gaussian mixture model; CLUSTER-FIRST (C1), which infer clusters in each cell type separately and uses post-processing to match the modules from one cell type to another. The higher the value the better the cluster quality. **B.** F-score (harmonic mean of precision and recall) to assess the reliability of the module transitions obtained from CLUSTER-FIRST and CMINT, when applied to simulated data with known transitions. The higher the value the better. Merge first does not infer any transitions and therefore is not shown in this figure. **C.** Precision (the fraction of predicted transitions that are correct) and **D.** Recall (the fraction of true transitions that are predicted) of module transition prediction using CMINT and CLUSTER-FIRST approach.

Supp Fig S1

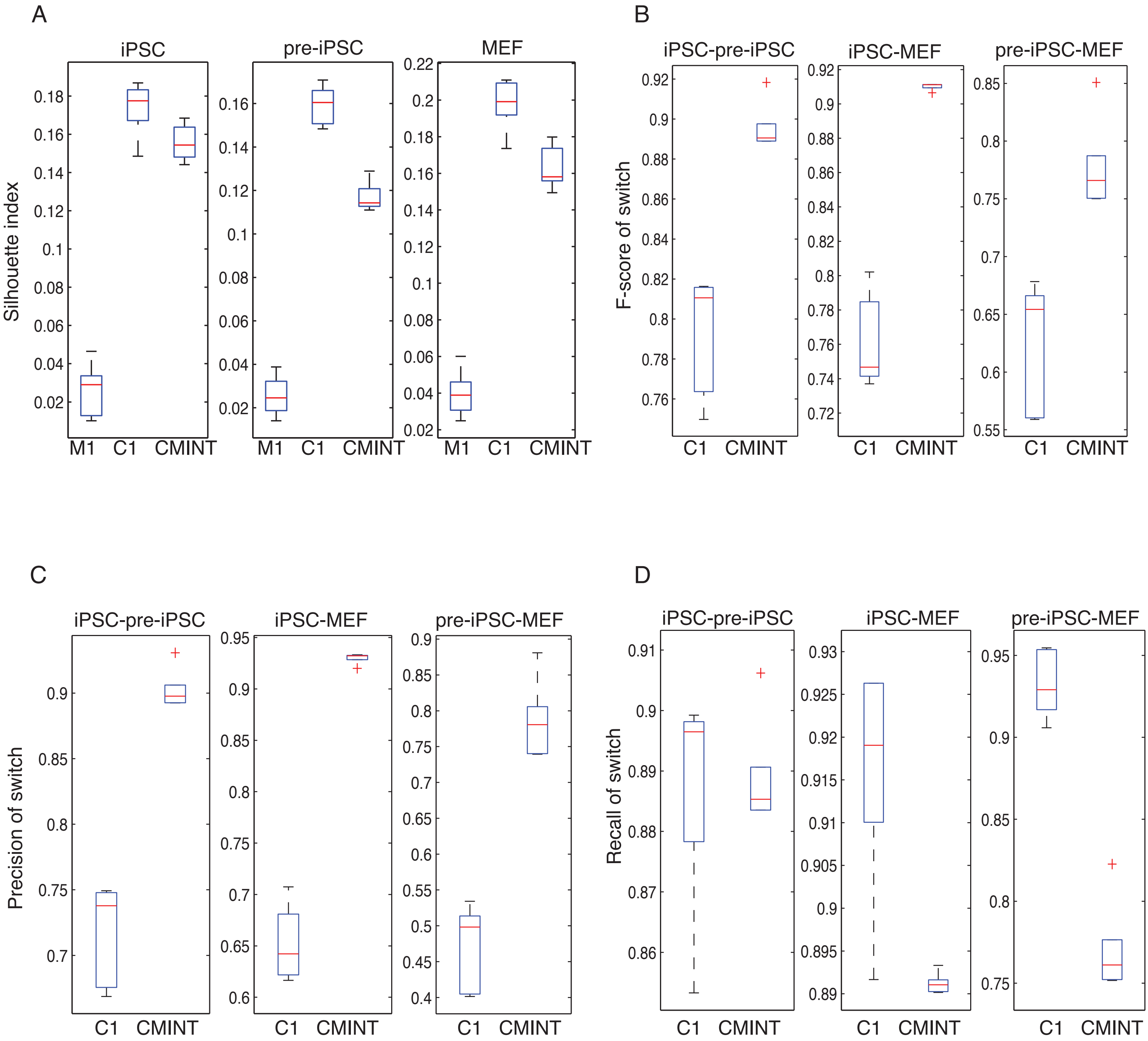
A
iPSC
pre-iPSC
MEF
Silhouette index
M1
C1
CMINT
B
iPSC-pre-iPSC
iPSC-MEF
pre-iPSC-MEF
F-score of switch
C
Precision of switch
D
Recall of switch
