# Supplementary material for: Chromatin module inference on cellular trajectories identifies key transition points and poised epigenetic states in diverse developmental processes
Source: Genome Res. 2017 Jul;27(7):1250–62. doi: 10.1101/gr.215004.116 (PMC5495076; doi:10.1101/gr.215004.116)

Supp Fig S2: Comparison of CMINT against ChromHMM on 15 cell types of the hematopoiesis lineage. **A.** Heatmaps of 16 chromatin modules obtained from ChromHMM applied to the hematopoietic system. Signal was calculated for each modification in 2000bp windows. Each row in each heatmap represents one region, each column represents one histone modification. Red: enriched, White: depleted. Height of each module is roughly proportional to the number of regions within it. **B.** Heatmaps of 16 CMINT modules when applied to 15 cell type data. This is the same result shown in **Supp Fig 5A** repeated for ease of comparison.

Supp Fig S2

A

ChromHMM clusters on the entire hematopoesis lineage

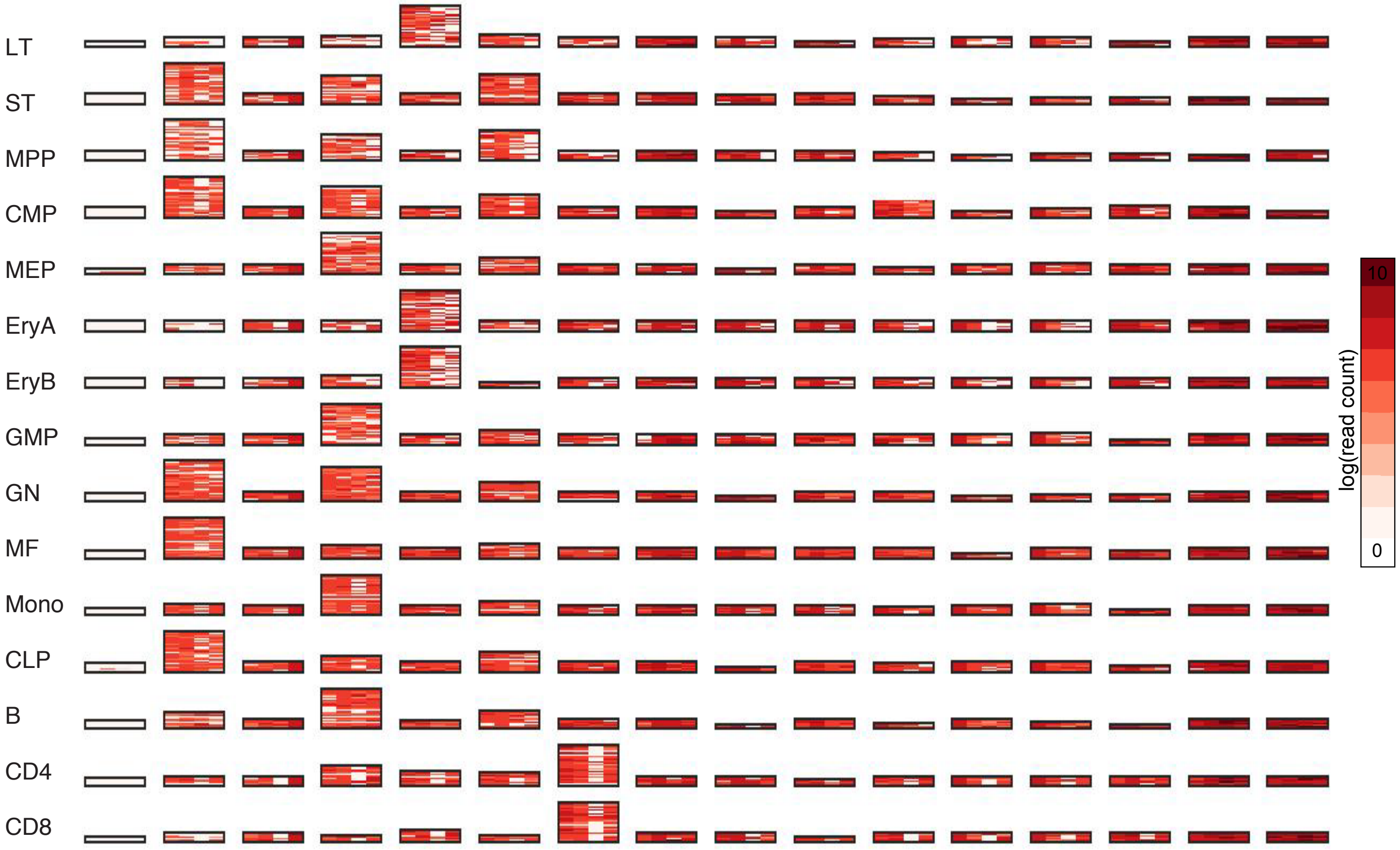

B

CMINT modules on the entire hematopoesis lineage

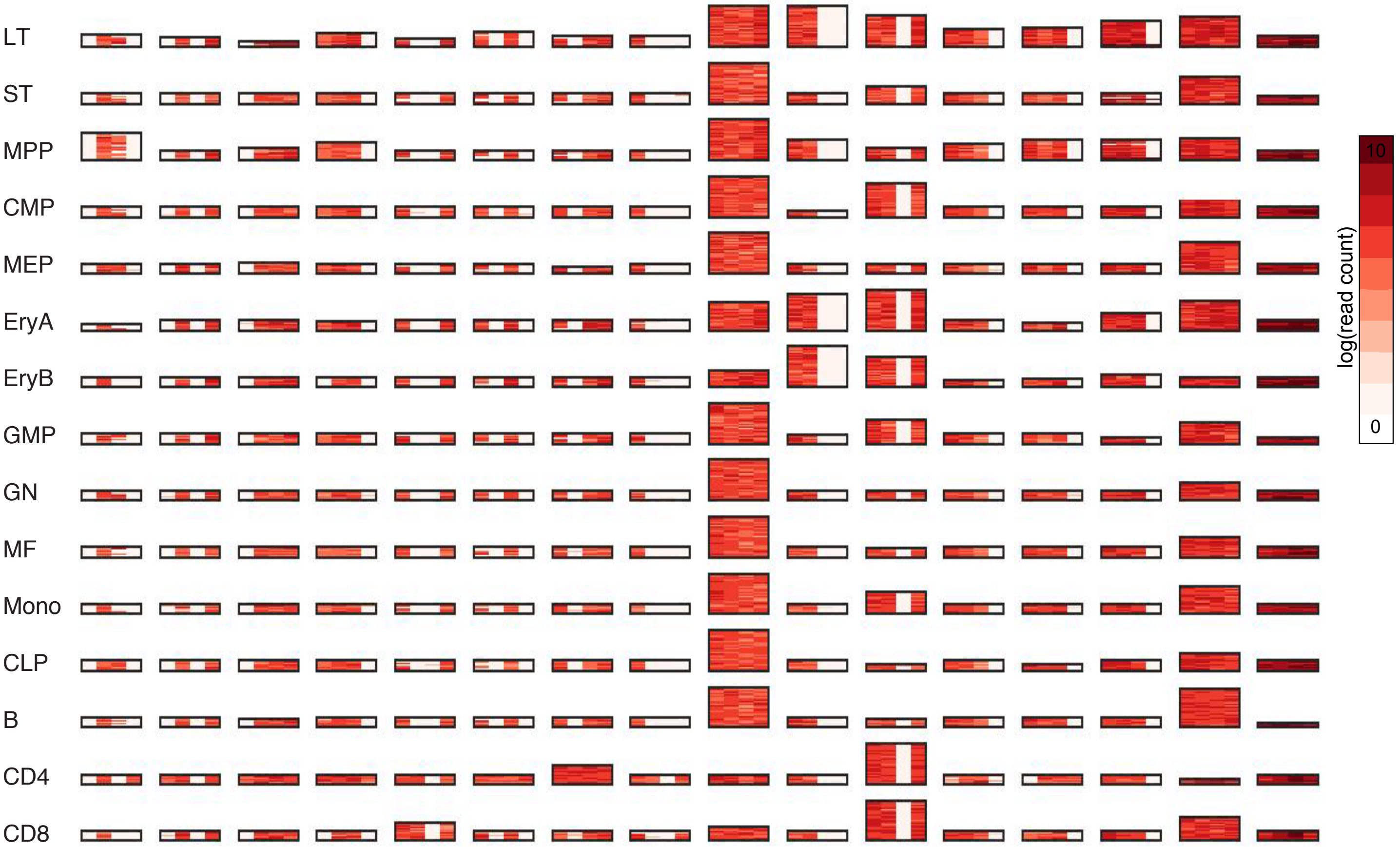

Supplement: Supplemental Material [file supp_gr.215004.116_Supplemental_Fig_S2.pdf]
