# Supplementary material for: Chromatin module inference on cellular trajectories identifies key transition points and poised epigenetic states in diverse developmental processes
Source: Genome Res. 2017 Jul;27(7):1250–62. doi: 10.1101/gr.215004.116 (PMC5495076; doi:10.1101/gr.215004.116)
Supplement: Supplemental Material [file supp_gr.215004.116_Supplemental_Fig_S3.pdf]

Supp Fig S3: Comparison of CMINT against GATE on seven cell types for the erythrocyte lineage. **A.** Heatmaps of 16 chromatin modules (major columns) obtained from GATE applied to seven cell types of erythrocyte lineage. Signal was calculated for each modification in 2000bp windows. Each row in each heatmap represents one region, each column represents one histone modification. Red: enriched, White: depleted. Height of each module is roughly proportional to the number of regions within it. The first row has empty clusters for GATE because GATE assumes that in the first time point (ST cell type) there is only one state. **B.** Heatmaps of 16 chromatin modules inferred by CMINT when applied to the erythrocyte lineage.

Supp Fig S3

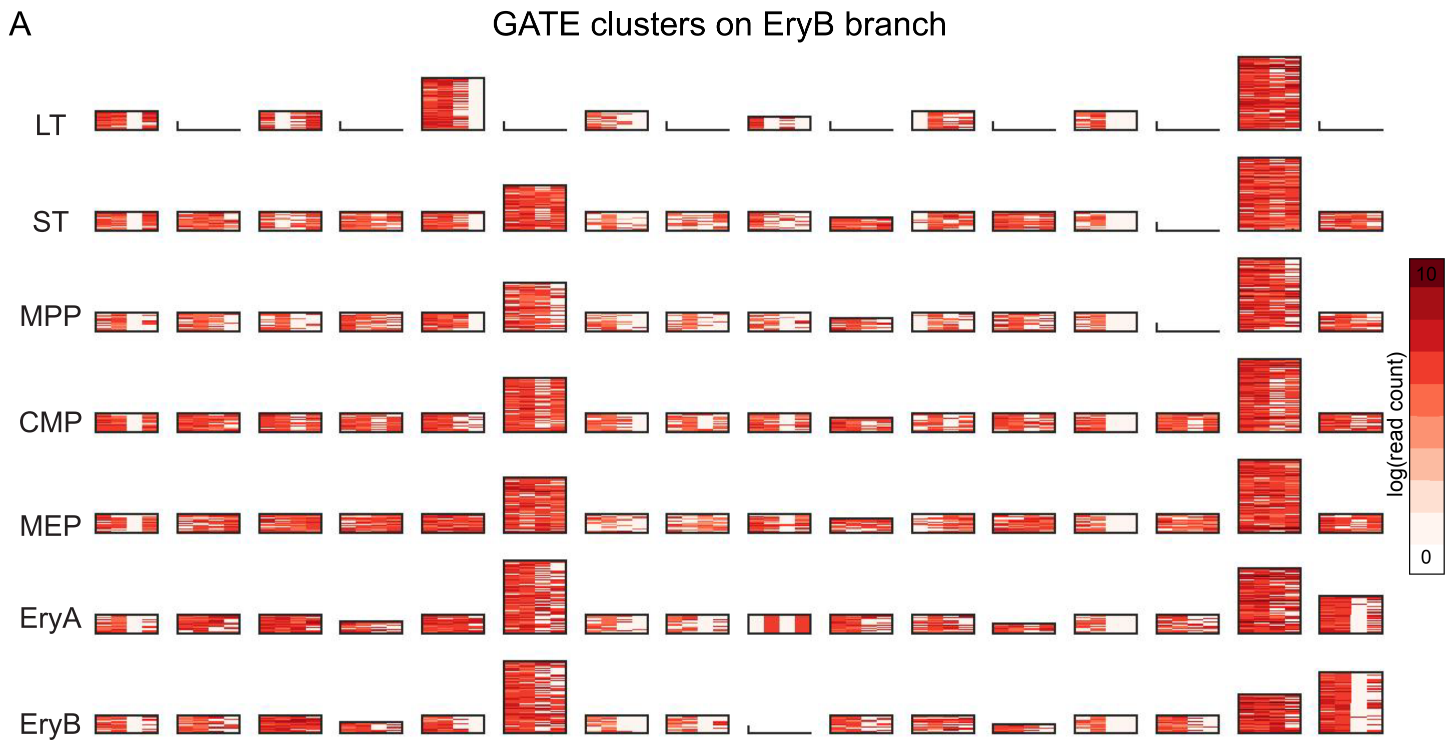
A
GATE clusters on EryB branch
LT
ST
MPP
CMP
MEP
EryA
EryB
10
log(read count)
0

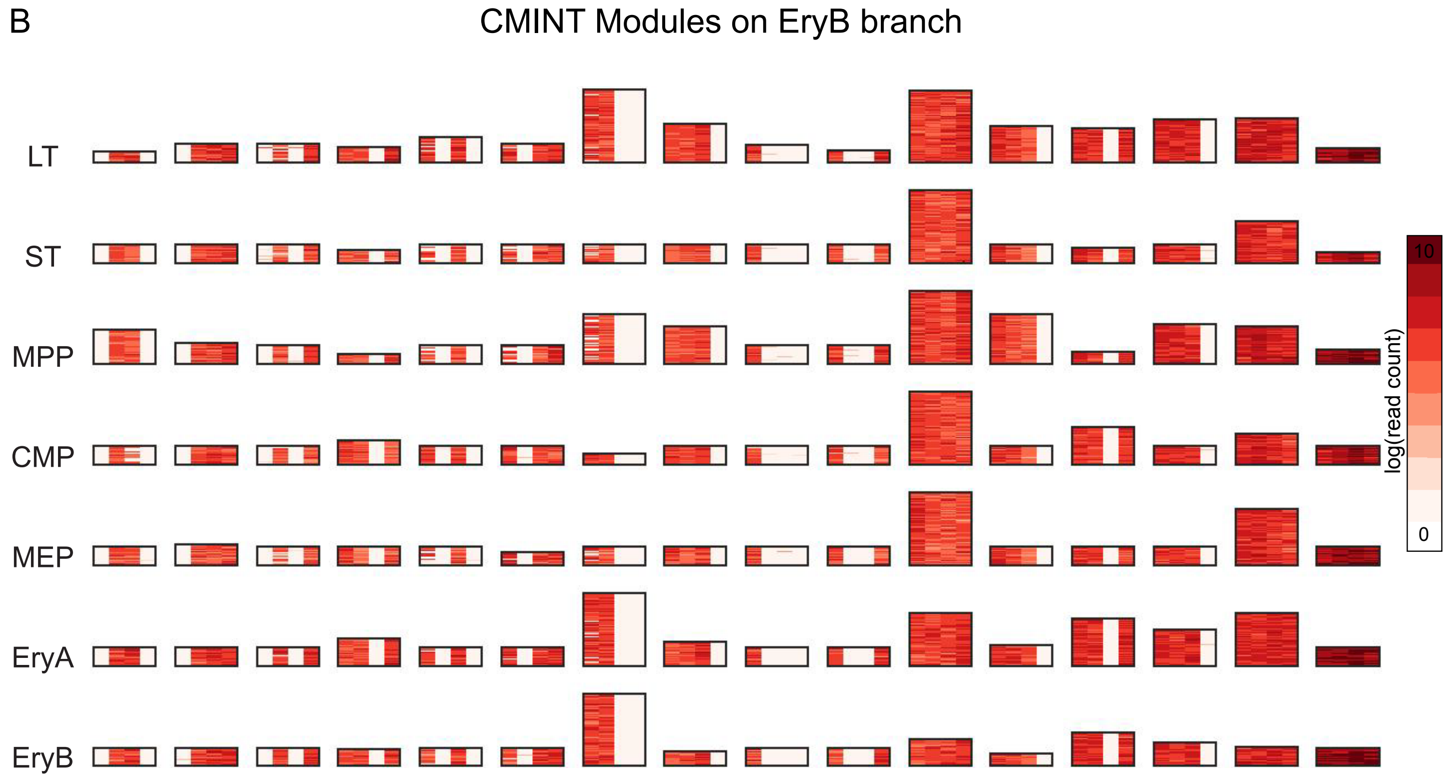
B
CMINT Modules on EryB branch
LT
ST
MPP
CMP
MEP
EryA
EryB
10
log(read count)
0
