# Supplementary material for: Chromatin module inference on cellular trajectories identifies key transition points and poised epigenetic states in diverse developmental processes
Source: Genome Res. 2017 Jul;27(7):1250–62. doi: 10.1101/gr.215004.116 (PMC5495076; doi:10.1101/gr.215004.116)
Supplement: Supplemental Material [file supp_gr.215004.116_Supplemental_Fig_S4.pdf]

Supp Fig S4: Using CMINT for further analysis of reprogramming cell types. **A.** Possible trajectories for the three the cell types. Data log likelihood value of CMINT models using different types of topologies. **B.** Modules obtained at optimal k values for each of the cell types. **C.** Box plots of gene expression of the chromatin modules in MEF (top) and pre-iPSC (bottom) for the modules shown in **Fig 3A**. **D.** Gene ontology enrichment for each of the modules in iPSC. The top two categories are listed along with the -log(p-value) of enrichment as scores.

Supp Fig S4

A

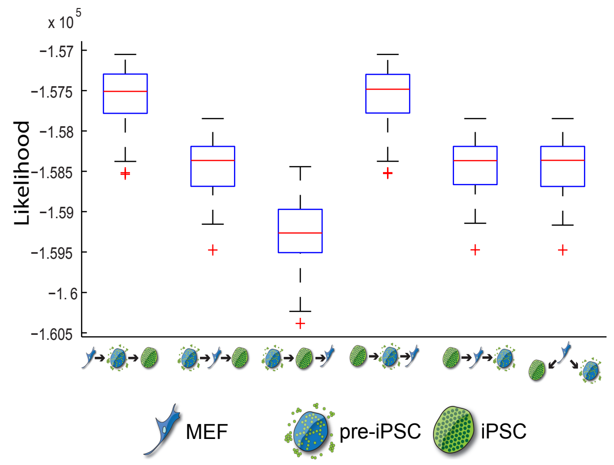

B

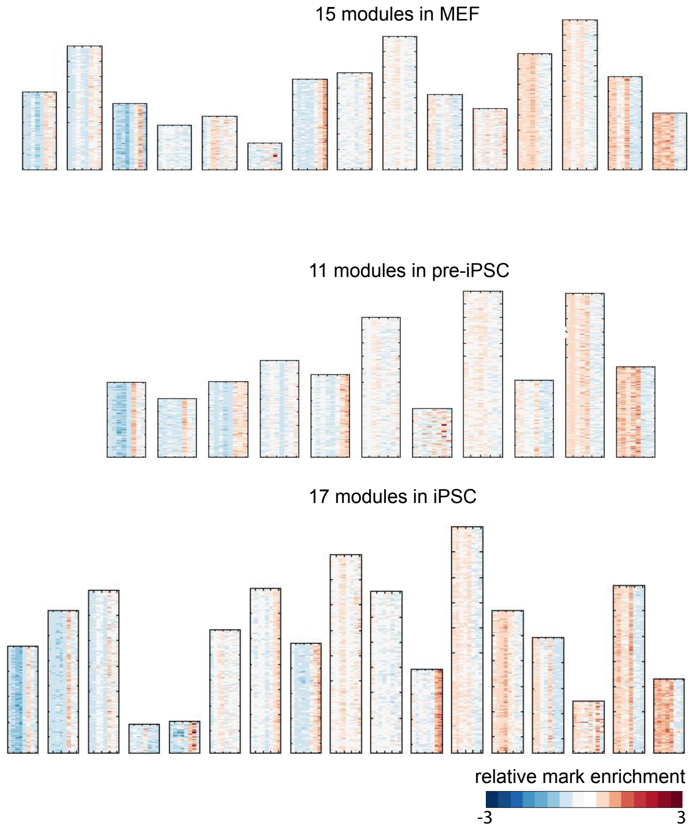

C

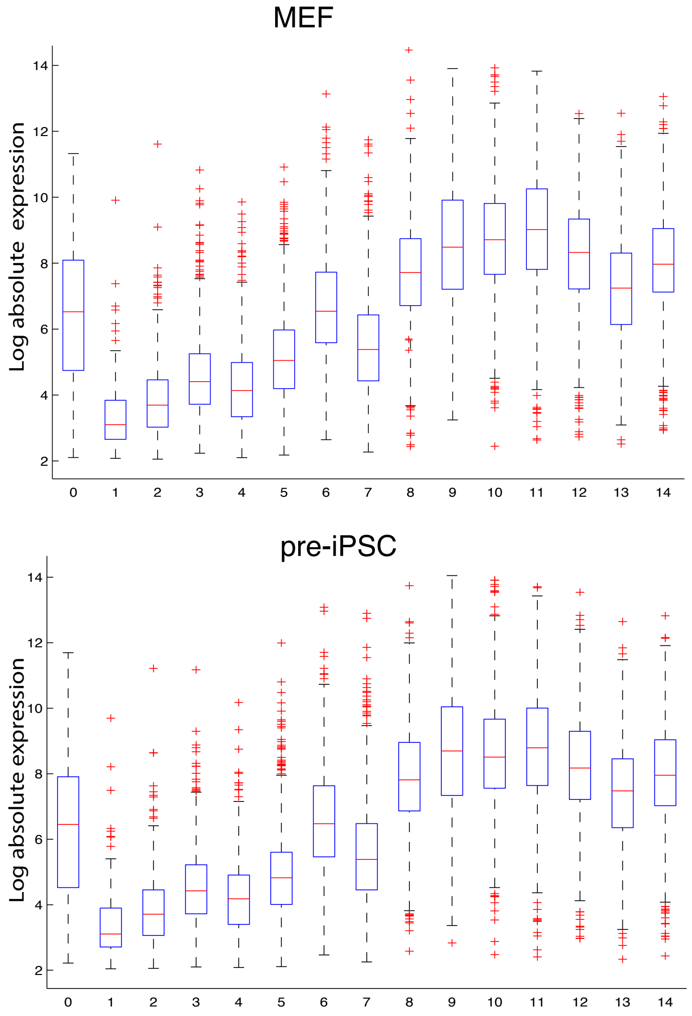

D

Module #	Ontology	Score
0	Cation binding	6.02
1	Sensory transduction	23.47
	Taste transduction	7.38
2	Receptor activity	38.33
	Inflammatory response	9.96
3	Serine hydroxylase activity	11.67
	Response to wounding	10.3
4	Transmembrane receptor activity	20.53
	Cell-cell adhesion	16.18
5	Transcription - morphogenesis	43.96
	Organ development	35.87
6	Regulation of cell communication	6.94
	Regulation of developmental process	6.78
7	Regulation of intracellular process	4.32
	Cell differentiation	3.5
8	Mitcohondrion related	4.67
	Transcription factor activty	3.63
9	Chromatin assembly	9.22
	Organ development	4.25
10	Protein metabolism- ubiquitination	10.22
	Transcription	9.81
11	Translation	28.48
	Gene expression	14.8
12	Protein Vesicle transport	11.32
	Dna repair	9.65
13	Mitochondrion related	8.98
	Metal binding	7.42
14	Biopolymer modification	13.79
	Kinase activity	5.55
