# Supplementary material for: Chromatin module inference on cellular trajectories identifies key transition points and poised epigenetic states in diverse developmental processes
Source: Genome Res. 2017 Jul;27(7):1250–62. doi: 10.1101/gr.215004.116 (PMC5495076; doi:10.1101/gr.215004.116)
Supplement: Supplemental Material [file supp_gr.215004.116_Supplemental_Fig_S5.pdf]

Supp Fig S5: CMINT chromatin modules identified in the hematopoiesis lineage. **A.** Heatmaps of 16 chromatin modules numbered from 0-15, obtained from CMINT applied to the hematopoietic system. Signal value for each mark was calculated for each modification in 2000bp windows. Each row in each heatmap represents 1 region each column represents one histone modification. Red: enriched, white: depleted. Height of each module is roughly proportional to the number of regions within it. **B.** ORegAnno cis-regulatory enrichment of selected factors based on enrichment in 12 out of the 15 cell types in specific modules on the left and for each cell type indicated on the top. The number next to each element is the module (in any cell type) that has the enrichment for the element. Color bar represents -log(p-value) of enrichment of binding sites in a particular module. Additional enrichments for individual modules are shown in **Supp Fig S8**.

Supp Fig S5

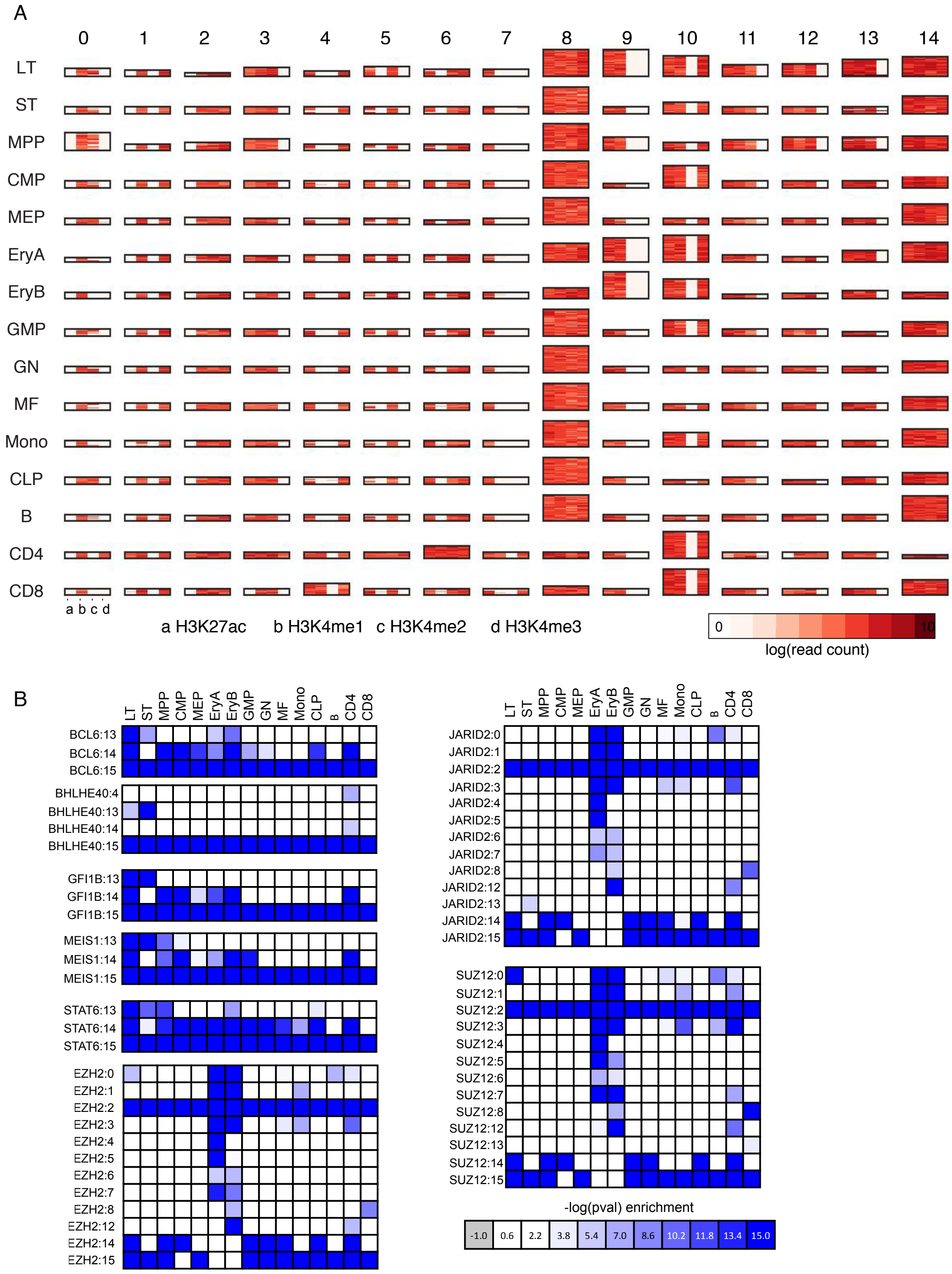
A
0 1 2 3 4 5 6 7 8 9 10 11 12 13 14 15
LT
ST
MPP
CMP
MEP
EryA
EryB
GMP
GN
MF
Mono
CLP
B
CD4
CD8
a b c d
a H3K27ac
b H3K4me1
c H3K4me2
d H3K4me3
0
10
log(read count)
B
LT ST MPP CMP MEP EryA EryB GMP GN MF Mono CLP B CD4 CD8
BCL6:13
BCL6:14
BCL6:15
BHLHE40:4
BHLHE40:13
BHLHE40:14
BHLHE40:15
GFI1B:13
GFI1B:14
GFI1B:15
MEIS1:13
MEIS1:14
MEIS1:15
STAT6:13
STAT6:14
STAT6:15
EZH2:0
EZH2:1
EZH2:2
EZH2:3
EZH2:4
EZH2:5
EZH2:6
EZH2:7
EZH2:8
EZH2:12
EZH2:14
EZH2:15
JARID2:0
JARID2:1
JARID2:2
JARID2:3
JARID2:4
JARID2:5
JARID2:6
JARID2:7
JARID2:8
JARID2:12
JARID2:13
JARID2:14
JARID2:15
SUZ12:0
SUZ12:1
SUZ12:2
SUZ12:3
SUZ12:4
SUZ12:5
SUZ12:6
SUZ12:7
SUZ12:8
SUZ12:12
SUZ12:13
SUZ12:14
SUZ12:15
-log(pval) enrichment
-1.0 0.6 2.2 3.8 5.4 7.0 8.6 10.2 11.8 13.4 15.0
