# Supplementary material for: Chromatin module inference on cellular trajectories identifies key transition points and poised epigenetic states in diverse developmental processes
Source: Genome Res. 2017 Jul;27(7):1250–62. doi: 10.1101/gr.215004.116 (PMC5495076; doi:10.1101/gr.215004.116)
Supplement: Supplemental Material [file supp_gr.215004.116_Supplemental_Fig_S6.pdf]

Supp Fig S6: Similarity of CMINT modules inferred from 1 million 2000 bp genomic regions in the hematopoietic lineage. Plot of similarity (F-score) of module membership of regions between each pair of cell types over the entire genome. Two different scales are used, Red: similarity for diagonal modules; Blue: similarity for off-diagonal modules. The more red or blue an entry, the more similar are the modules. An example pairwise comparison is highlighted for the MF and GMP cell types.

Supp Fig S6

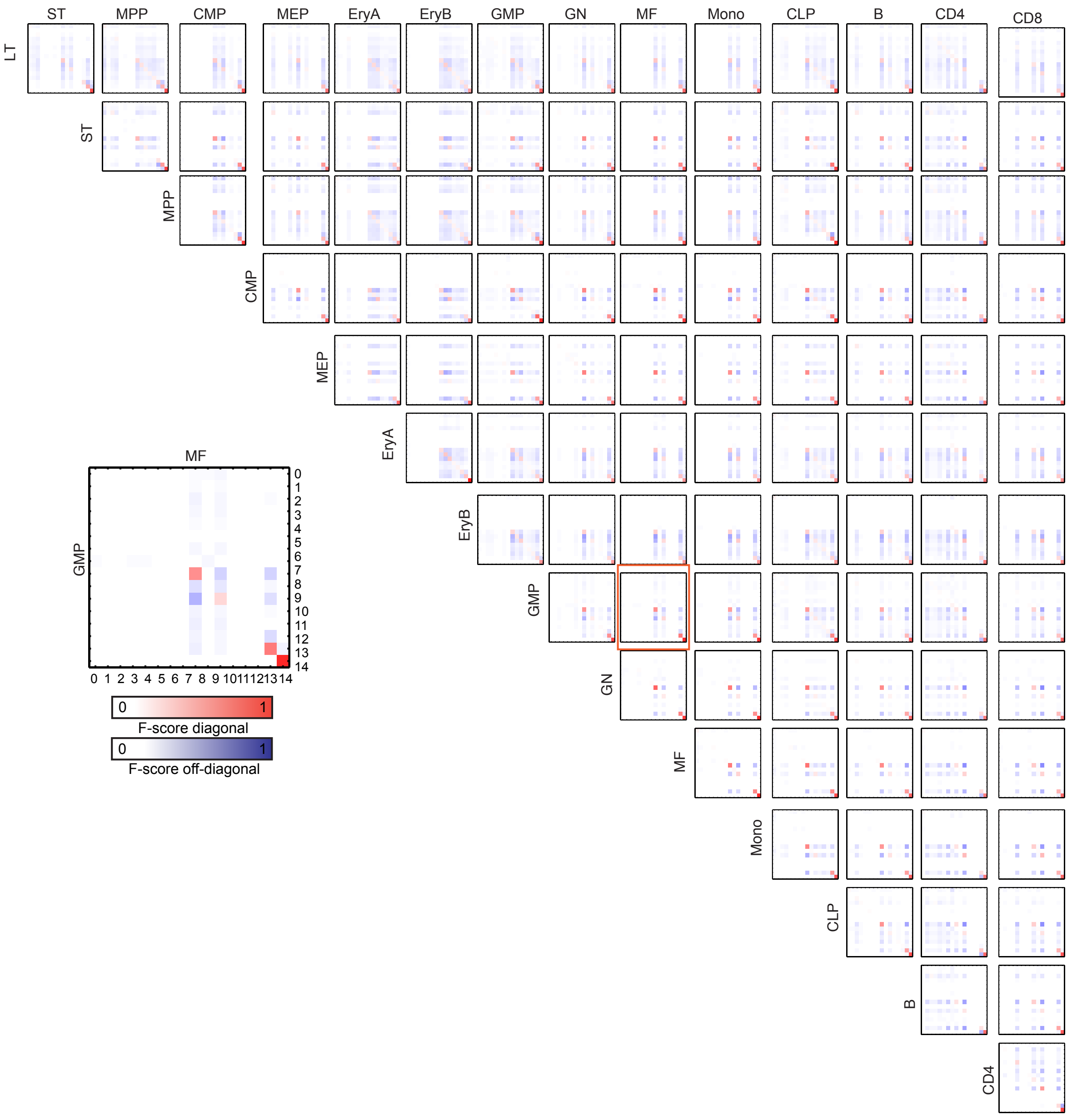

ST
MPP
CMP
MEP
EryA
EryB
GMP
GN
MF
Mono
CLP
B
CD4
CD8
LT
ST
MPP
CMP
MEP
EryA
EryB
GMP
GN
MF
Mono
CLP
B
CD4
MF
GMP
0 1 2 3 4 5 6 7 8 9 10 11 12 13 14
0
1
F-score diagonal
0
1
F-score off-diagonal
