# Supplementary material for: Chromatin module inference on cellular trajectories identifies key transition points and poised epigenetic states in diverse developmental processes
Source: Genome Res. 2017 Jul;27(7):1250–62. doi: 10.1101/gr.215004.116 (PMC5495076; doi:10.1101/gr.215004.116)
Supplement: Supplemental Material [file supp_gr.215004.116_Supplemental_Fig_S7.pdf]

Supp Fig S7: Significance of similarity of CMINT modules inferred from 1 million 2000 bp genomic regions of the hematopoietic lineage. Shown are the significance of similarity of pairs of modules for pairs of cell types in the hematopoiesis lineage. Similar to **Supp Fig S6**, but using the negative log of p-value of the overlap significance (computed similarly to **Fig 4A**). Two different scales are used, Red: similarity for diagonal modules; Blue: similarity for off-diagonal modules. The more red or blue an entry, the more significant is the similarity of the corresponding modules.

Supp Fig S7

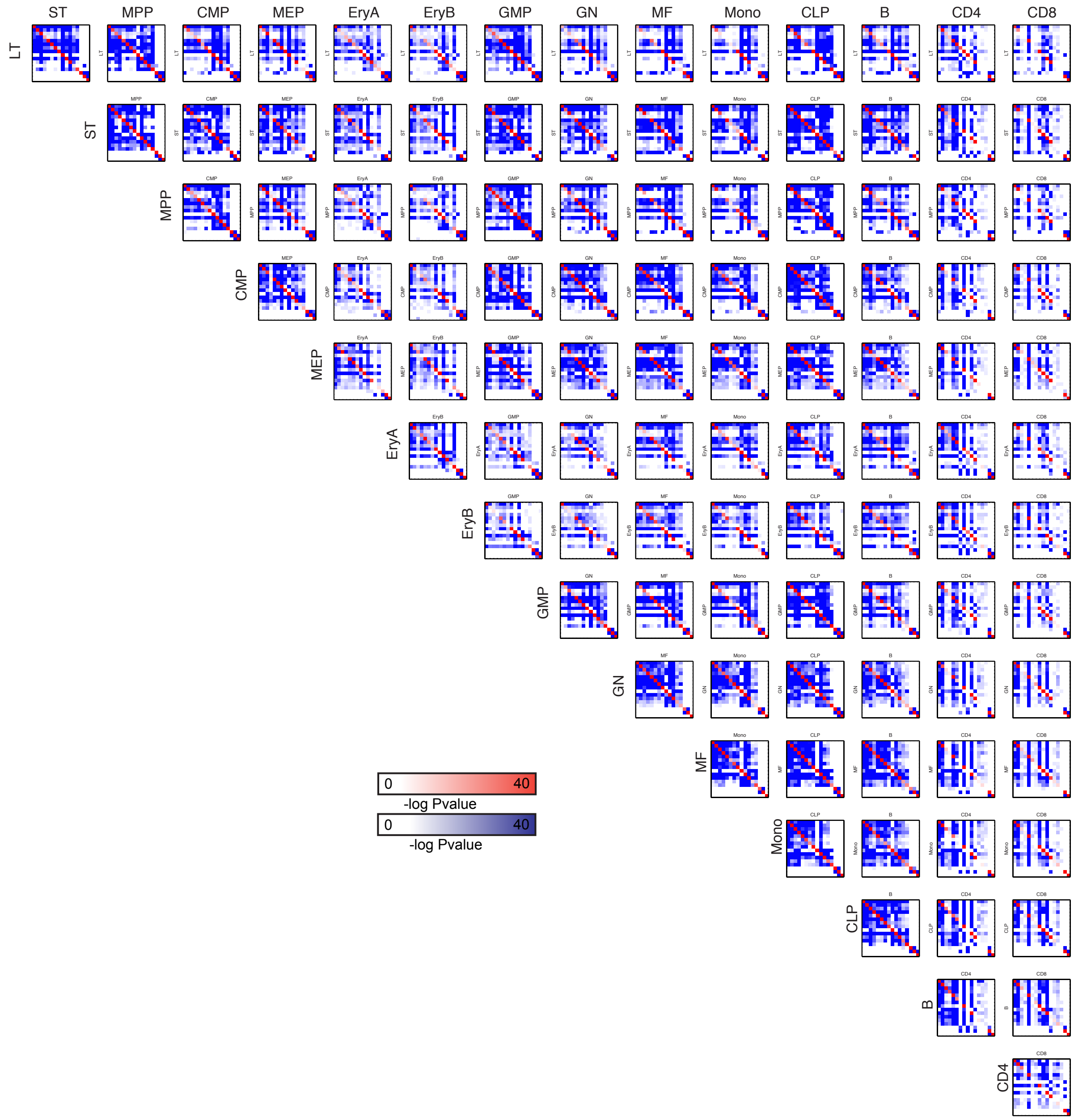

ST
MPP
CMP
MEP
EryA
EryB
GMP
GN
MF
Mono
CLP
B
CD4
CD8
LT
0
40
-log Pvalue
0
40
-log Pvalue
