# Supplementary material for: Chromatin module inference on cellular trajectories identifies key transition points and poised epigenetic states in diverse developmental processes
Source: Genome Res. 2017 Jul;27(7):1250–62. doi: 10.1101/gr.215004.116 (PMC5495076; doi:10.1101/gr.215004.116)
Supplement: Supplemental Material [file supp_gr.215004.116_Supplemental_Fig_S8.pdf]

Supp Fig S8: Enrichment of ORegAnno regulatory elements across multiple cell types in CMINT modules inferred from 1 million 2000bp genomic regions. Shown for each module are the ORegAnno elements enriched in a module in any of the cell types. The intensity of blue is proportional to -log(p-value) of enrichment (FDR corrected Hyper-geometric test) of elements in these modules. The elements are grouped based on the module they are enriched in. Only elements not shown in **Supp Fig S5B** are shown. Two versions of ATOH1 were available in the ORegAnno database. ATOH1v1 corresponds to the ATOH1 ORegAnno element and ATOH1v2 corresponds to the Atoh1 ORegAnno element.

Supp Fig S8

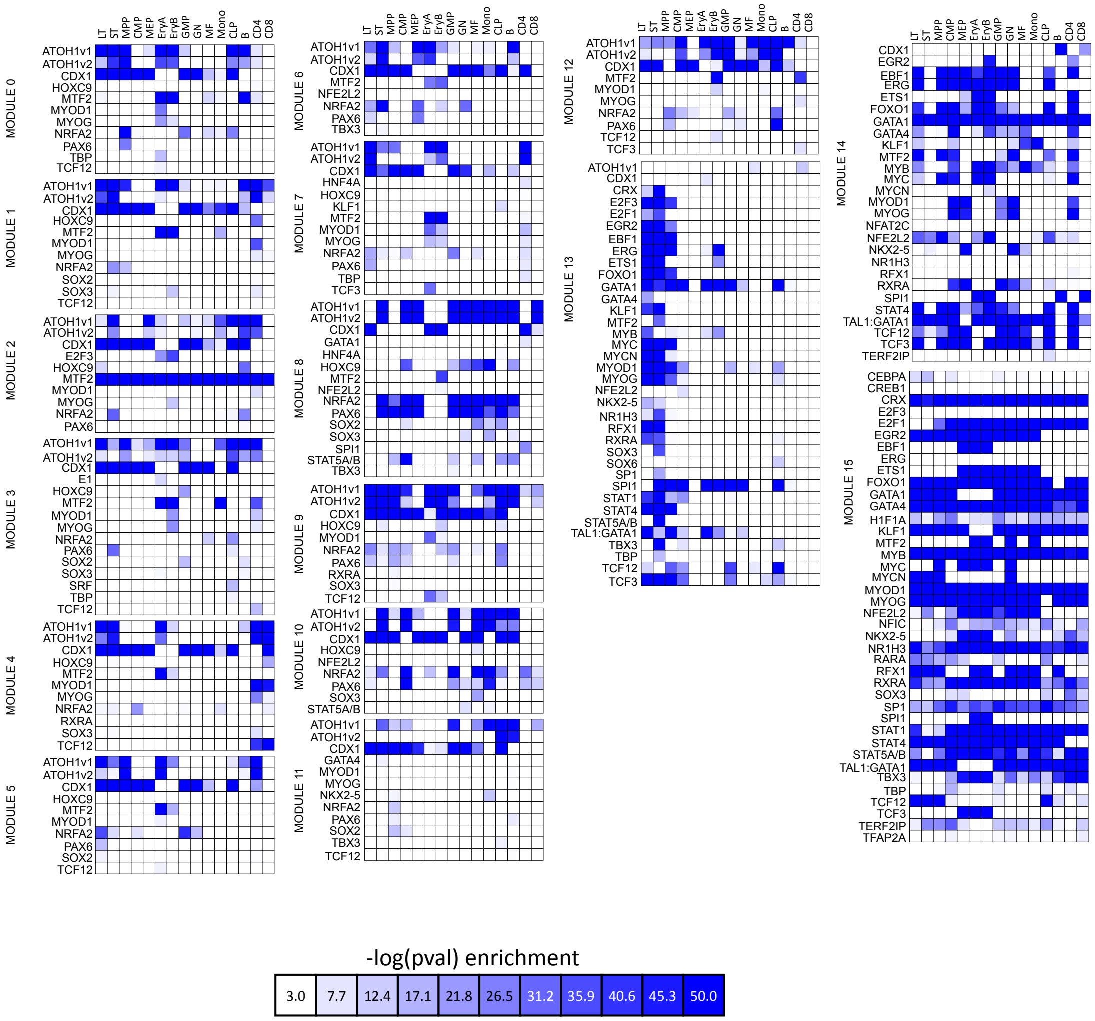
LT ST MPP CMP MEP EryA EryB GMP GN MF Mono CLP B CD4 CD8
MODULE 0
MODULE 1
MODULE 2
MODULE 3
MODULE 4
MODULE 5
MODULE 6
MODULE 7
MODULE 8
MODULE 9
MODULE 10
MODULE 11
MODULE 12
MODULE 13
MODULE 14
MODULE 15
-log(pval) enrichment
3.0 7.7 12.4 17.1 21.8 26.5 31.2 35.9 40.6 45.3 50.0
