# Supplementary material for: Chromatin module inference on cellular trajectories identifies key transition points and poised epigenetic states in diverse developmental processes
Source: Genome Res. 2017 Jul;27(7):1250–62. doi: 10.1101/gr.215004.116 (PMC5495076; doi:10.1101/gr.215004.116)
Supplement: Supplemental Material [file supp_gr.215004.116_Supplemental_Fig_S9.pdf]

Supp Fig S9: Significance of similarity of CMINT modules inferred from $\sim$28,000 2000 bp genomic regions in the hematopoietic lineage. Shown are the significance of similarity of pairs of modules for pairs of cell types in the hematopoiesis lineage. Similar to **Supp Fig S7**. Two different scales are used, Red: similarity for diagonal modules; Blue: similarity for off-diagonal modules. The more red or blue an entry, the more significant is the similarity of modules.

Supp Fig S9

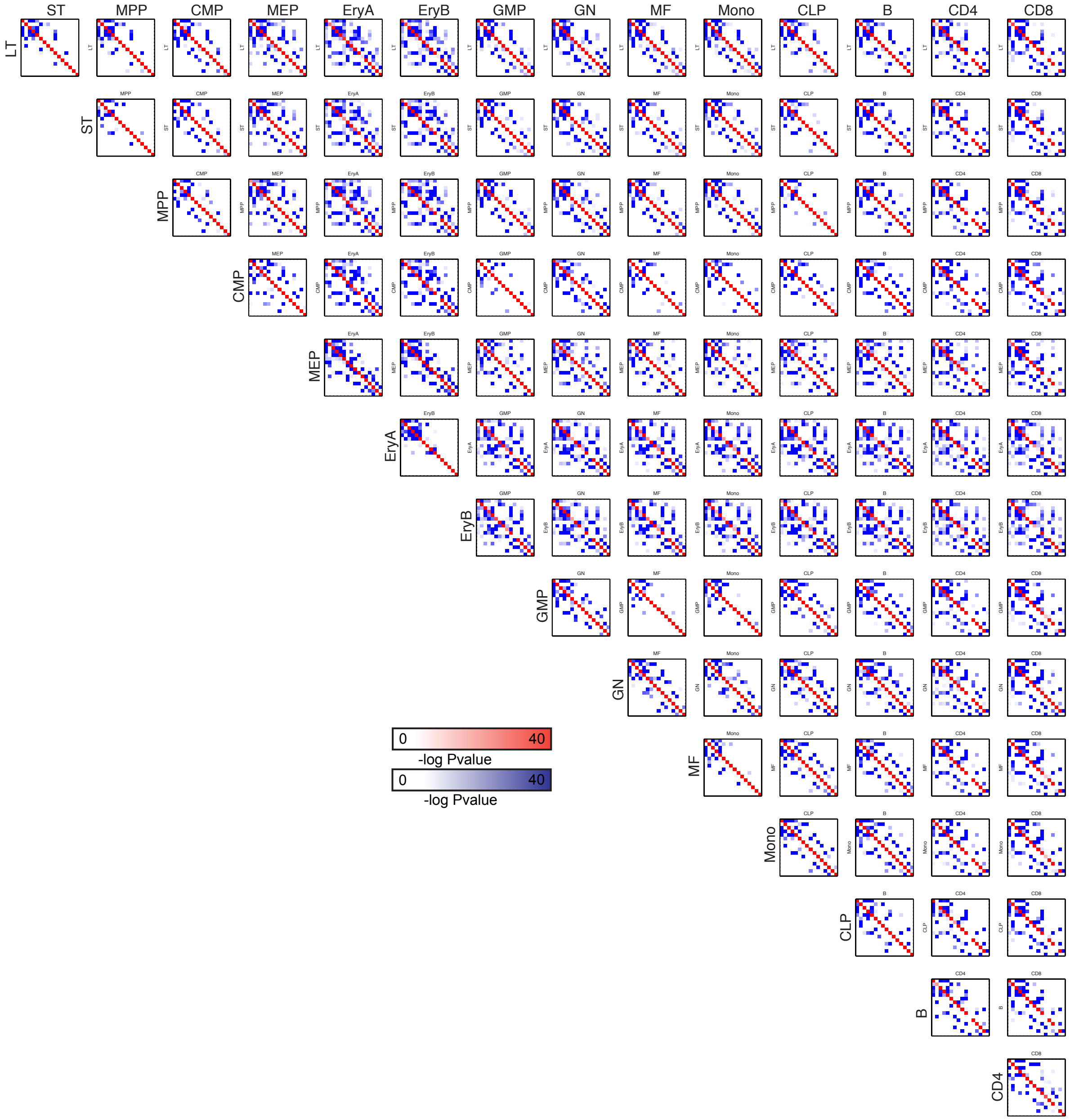
ST
MPP
CMP
MEP
EryA
EryB
GMP
GN
MF
Mono
CLP
B
CD4
CD8
LT
0
40
-log Pvalue
0
40
-log Pvalue
