# Supplementary material for: Chromatin module inference on cellular trajectories identifies key transition points and poised epigenetic states in diverse developmental processes
Source: Genome Res. 2017 Jul;27(7):1250–62. doi: 10.1101/gr.215004.116 (PMC5495076; doi:10.1101/gr.215004.116)
Supplement: Supplemental Material [file supp_gr.215004.116_Supplemental_Fig_S10.pdf]

Supp Fig S10: Alternative tree structures for the hematopoietic lineage. Eight possible trajectories for the 15 cell types of the hematopoiesis lineage shown on the x-axis, Data log likelihood value of CMINT models obtained on different types of topologies are presented on the y-axis.

Supp Fig S10

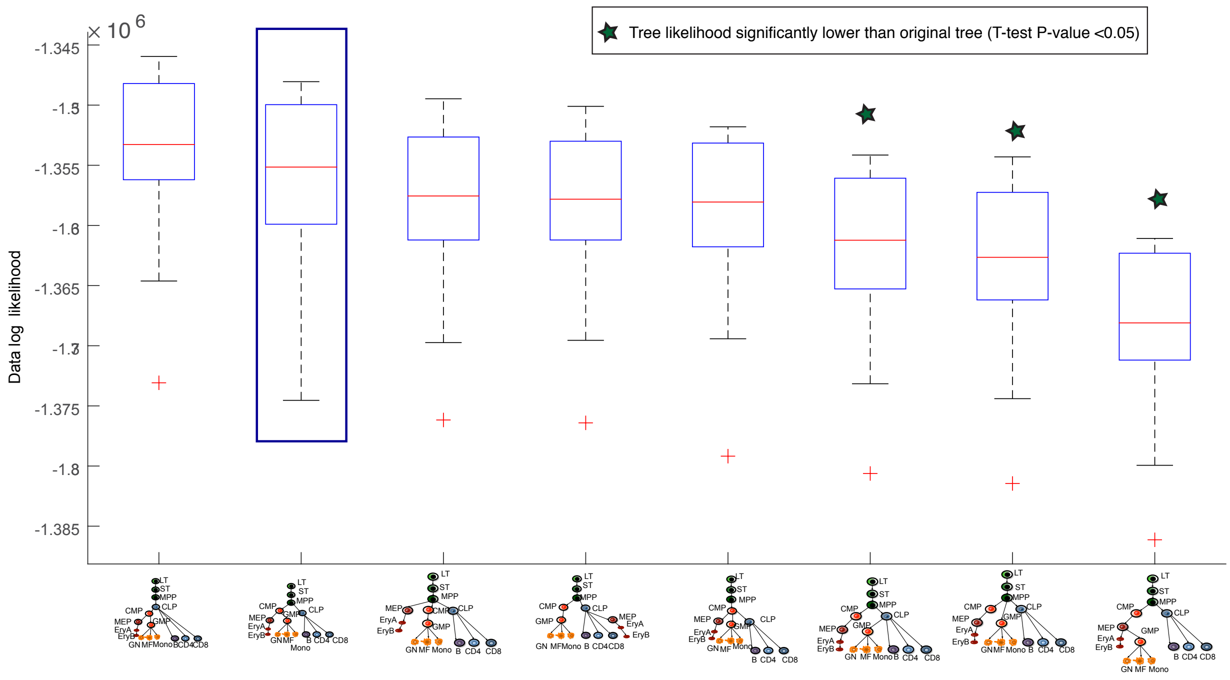

Tree likelihood significantly lower than original tree (T-test P-value <0.05)
Data log likelihood
× 10^6
-1.345
-1.355
-1.365
-1.375
-1.385
