# Supplementary material for: Chromatin module inference on cellular trajectories identifies key transition points and poised epigenetic states in diverse developmental processes
Source: Genome Res. 2017 Jul;27(7):1250–62. doi: 10.1101/gr.215004.116 (PMC5495076; doi:10.1101/gr.215004.116)
Supplement: Supplemental Material [file supp_gr.215004.116_Supplemental_Fig_S11.pdf]

Supp Fig S11: Determining optimal number of clusters for each cell type. Shown are the penalized test data likelihood as a function of increasing number of modules for the three reprogramming cell types.

Supp Fig S11

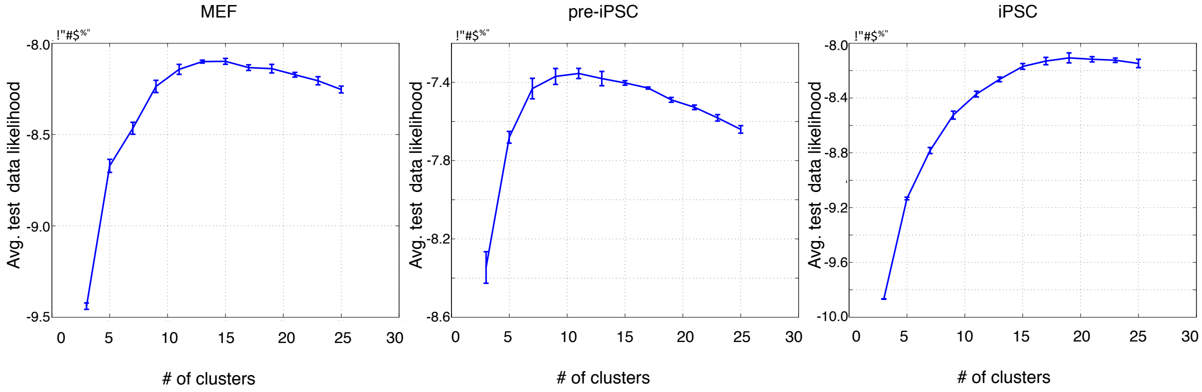
MEF
pre-iPSC
iPSC
Avg. test data likelihood
# of clusters
-8.0
-8.5
-9.0
-9.5
-7.4
-7.8
-8.2
-8.6
-8.4
-8.8
-9.2
-9.6
-10.0
0
5
10
15
20
25
30
