# Supplementary material for: Chromatin module inference on cellular trajectories identifies key transition points and poised epigenetic states in diverse developmental processes
Source: Genome Res. 2017 Jul;27(7):1250–62. doi: 10.1101/gr.215004.116 (PMC5495076; doi:10.1101/gr.215004.116)
Supplement: Supplemental Material [file supp_gr.215004.116_Supplemental_Methods.pdf]

Supplemental Methods

Details of Chromatin Module INference on Trees (CMINT)

The CMINT framework aims to solve multiple clustering problems, one per cell type, simultaneously, by using a probabilistic generative model of the data. The clustering per dataset is based on a Gaussian mixture model (GMM) (Hastie et al., 2003). CMINT is similar to the Arboretum approach we developed for clustering multiple species (Roy et al., 2013), which we extend here to be applicable to cell lineages. Specifically to handle cell lineage data: (a) we handle arbitrary tree topologies unlike in Arboretum which can handle only a binary tree, (b) we extend the generative model to handle the observed data at the leaf and internal nodes of the tree. Below we describe the key aspects of the CMINT model and learning algorithm to estimate the parameters of this model.

CMINT generative model: The generative model must generate values for two types of random variables: (a) hidden variables representing the cluster assignments, and (b) observed variables encoding expression for each gene at all points in the tree. Let n denote the number of different chromatin mark datasets and τ denote the lineage tree describing how the cell types are related. Let k be the number of clusters for each cell type's chromatin mark dataset. The cluster assignments are matched between the datasets by the generative model of cluster assignments. CMINT's generative model is defined by the following components: $\{\mathbf{M}, \mathbf{S}, \pi, \mathbf{T}\}$, where $\mathbf{M} = \{\boldsymbol{\mu}_1, \cdots, \boldsymbol{\mu}_l, \cdots, \boldsymbol{\mu}_n\}$ and $\mathbf{S} = \{\boldsymbol{\Sigma}_1, \cdots, \boldsymbol{\Sigma}_l, \cdots, \boldsymbol{\Sigma}_n\}$ correspond to the mean and covariances associated with the n cell type-specific chromatin mark datasets. π denotes a multinomial distribution for cluster assignments and $\mathbf{T}$ is the set of transition probability matrices for each branch on the tree defining the probability of genomic region to maintain or change its module assignment from its immediate predecessor cell state. Each $\boldsymbol{\mu}_l$ and $\boldsymbol{\Sigma}_l$ corresponds to the parameters defining the

Gaussian mixture model for the lth cell type, including k mean vectors $\boldsymbol{\mu}_l = \{\mu_{l1}, \cdots, \mu_{lk}\}$ and k covariances $\boldsymbol{\Sigma} = \{\Sigma_{l1}, \cdots, \Sigma_{lk}\}$ for the k Gaussian components of a GMM. Each transition probability matrix T_l for a cell type l that is not the root, is $k \times k$ where the entry $T_l(i, j)$ specifies the probability of a region to be in cluster i in node l of the tree, given that in its parent the gene is in cluster j. The generative model of CMINT generates the cluster assignments and chromatin profiles for each genomic region g in all n datasets in the following manner. We use l to denote the lth node in the tree and $p(l)$ as the parent of node l. Let r denote the index of the root node.

- Set current node l to root, r.
- For each node l on tree τ
- if l is the root node, draw a cluster assignment for genomic region g by sampling from π, $k_g^l \sim \pi$
- else
  - j be the g's cluster assignment in l's parent
  - $k_g^c \sim T_l(j, :)$
  - Draw chromatin profile x_g^l for g in l using μ_{lk}, Σ_{lk}, where $k = k_g^l$.

At the end of this process, we have the chromatin profile for region g at each point in the tree τ. The CMINT algorithm uses the Expectation Maximization (EM) framework to infer all parameters. In the E-step, we infer the expected values of cluster assignments, $\gamma_{k|k'}^{li}$ of all genes at all the leaf nodes. We then recursively infer the expected values of cluster assignments of an intermediate node using the data nodes in the subtree below it and at this node. In the M-step, we estimate the mean and co-variance matrix of each cluster using $\gamma_{k|k'}^{li}$ for dataset c and also the transition probabilities. These steps are described below.

Expectation step: To infer the probability of a module assignment, we define k to be cluster assignment at one node and k' to be cluster assignment at the parent node. $\gamma_{k|k'}^{li}$ represents for posterior probability of the ith genomic region at the lth cell type to be in cluster k given that it is in cluster k' in the parent cell type of c. This will infer a $K \times K$ matrix $\Gamma^c(k, k') = \gamma_{k|k'}^{li}$. Then we infer in a recursive manner the posterior probabilities for all the intermediate nodes.

In addition to this we also used α^{li} which is $K \times 1$ vector, with each element representing $\alpha^{li}(k') = \sum_k \gamma^{li}_{k|k'}$ which says the probability of an observation given that the parent is in state k'. The estimation is done recursively as follows for a region i at node l

- if l is a leaf

  1. $\gamma^{li}_{k|k'} = P_l(k|k')e_{i|\mu^l_k,\sigma^l_k}$
  2. $\alpha^{li}(k') = \sum_k \gamma^{li}_{k|k'}$
  3. $\gamma^{li}_{k|k'} = \frac{\gamma^{li}_{k|k'}}{\alpha^{li}(k')}$

  where $P_l(k|k')$ is the conditional transition probability associated with cell type l. $e_{i|\mu^l_k,\sigma^l_k}$ corresponds to the probability of observing the ith measurement from the kth Gaussian component in cell type l.

- otherwise

  1. Estimate $\gamma^{l_c i}_{k''|k'}$ and $\alpha^{l_c i}$, for each child node l_c of l.
  2. $\gamma^{li}_{k|k'} = e_{i|\mu^l_k,\sigma^l_k} P_l(k|k') \prod_{l_c} \alpha^{l_c i}(k)$
  3. $\alpha^{li}(k') = \sum_k \gamma^{li}_{k|k'}$

Maximization step: There are two sets of parameters in this model: (a) cluster transition probabilities, (b) Gaussian mixture model parameters. We assume that the co-variance matrix is a diagonal matrix. Each of these parameters can be estimated in closed form by deriving the expected likelihood with respect to the parameters. Specifically, the mean μ^l_k is estimated as

$$\mu^l_k = \frac{\sum_i \mathbf{x}_i \alpha^{li}(k)}{\sum_i \alpha^{li}(k)}$$

Variance for the mth dimension is estimated as

$$\Sigma^l_k(m,m) = \frac{\sum_i (\mathbf{x}_i(m) - \mu^l_k(m))^2 \alpha^{li}(k)}{\sum_i \alpha^{li}(k)}$$

Transition probability for the lth cell type is

$$T_l(k|k') = \frac{\sum_i \gamma^{li}_{k|k'}}{\sum_{k,k'} \gamma^{li}_{k|k'}}$$

CMINT model learning: We begin with an initial clustering assignment obtained from partitioning genes into k partitions, where k specifies the number of clusters. This partitioning can be obtained by randomly splitting the data, or by a clustering algorithm. We next repeat the expectation and maximization steps until convergence or until a fixed number of iterations have been executed.

References

T. Hastie, R. Tibshirani, and J. H. Friedman. *The Elements of Statistical Learning*. Springer, second edition, July 2003. ISBN 0387952845.

Sushmita Roy, Ilan Wapinski, Jenna Pfiffner, Courtney French, Amanda Socha, Jay Konieczka, Naomi Habib, Manolis Kellis, Dawn Thompson, and Aviv Regev. Arboretum: reconstruction and analysis of the evolutionary history of condition-specific transcriptional modules. *Genome Research*, 23(6):1039–1050, 2013.

Details of ChIP-chip experiments

ChIP-chip experiments were performed exactly as described in Sridharan et. al 2009 (Sridharan et al. 2009). Briefly, one fully reprogrammed iPSC line and a partially reprogrammed line pre-iPSC line have been previously described and were derived by the introduction of retrovirally encoded *Pou5f1, Sox2, c-Myc* and *Klf4* into mouse embryonic fibroblasts that contained a Nanog-GFP reporter. After crosslinking with formaldehyde for 10 mins, cells were sonicated to achieve 300-500bp size. Data for H3K4me3 and H3K27me3 for the iPSC and pre-iPSC line has been previously published (Sridharan et al. 2009; Maherali et al. 2007). About 400-500 ug of chromatin was IPed with 10ug of purified antibody or 10ul of serum. Antibodies used were H3K4me3 Abcam ab8580, H3K27me3 Millipore 07-449– H3K9ac, H3K14ac and H3K18ac were kind gifts of Prof. Michael Grunstein at UCLA, H3K9me2 Abcam ab1220, H3K9me3 Abcam ab8898, H3K79me2 Activ motif- 39143. Elutes were amplified using the Sigma WGA kit and applied to Agilent mouse promoter array (G4490) according to the manufacturers instructions. Average probe signals were initially extracted in a 500bp window-step-wise manner as described previously (Maherali et al. 2007), and then averaged across the entire 8000bp region and used as input for the CMINT algorithm.

Determining the number of modules

For the reprogramming study we used a two-step procedure to determine the number of modules, k. First we used five-fold cross validation to determine the best k from the range of 3 to 25 in increments of 2, in each cell type as follows. For each k, we divided the chromatin mark data into five sets and learned a Gaussian Mixture Model (GMM) using four out of the five sets and estimated the likelihood of the left of out test set using the learned Gaussian mixture model. We added an MDL penalty to the likelihood of the test data to also account for model complexity. We repeated this procedure for each of the five sets of data, and computed the average test likelihood for each k. We plotted the test data likelihood as a function of k,

and found that the optimal value of k in each cell type asymptotes around k=15 (MEF was k=15, iPSC was k=17, and pre-iPSC was k=11). We selected k=15 as the best as it represented the average of the number of clusters. In addition, we also examined CMINT modules for k=20, 25, and 30 and did not find any increase in the number of patterns detected.

For the hematopoietic study we used ChromHMM to determine the optimal number of modules. We also experimented with higher number of modules with CMINT, but did not find dramatically different patterns in each cell type.

Comparison of CMINT against existing multi-cell type chromatin analysis tools

We compared CMINT against two existing tools that can handle multiple chromatin marks from multiple cell types: ChromHMM (Ernst & Kellis 2010) and GATE (Yu et al. 2013) using the 15 cell type chromatin mark data from Lara-Astiaso et al. (Lara-Astiaso et al. 2014). ChromHMM is a hidden Markov model based approach for performing unsupervised segmentation of genomic regions based on combinations of chromatin marks. ChromHMM learns a single hidden Markov model for genomic data from all cell types, followed by applying the HMM state inference to each cell type separately to perform genome segmentation. We applied ChromHMM to 15 cell type data to learn 16 different chromatin states at 2000bp resolution in each cell type. We determined 16 to be the optimal number of modules based on the ChromHMM algorithm. The GATE approach is based on a finite mixture model of chromatin mark profiles where each component of the mixture is a hidden Markov model. GATE assumes that each state of the hidden Markov model has two states which while inferring 8 clusters, would result in a total of 16 clusters for all cell types but the starting cell type. We compared the results of GATE and ChromHMM against CMINT using two cluster quality metrics: (a) Silhouette index, (b) Cluster coherence. The Silhouette index is a number between -1 and 1, the higher the value the better the clusters and is low both for over clustering or under clustering the data. We defined cluster coherence as the fraction of regions

with a Pearson's correlation of 0.8 or higher to the cluster mean. The higher the value the better the coherence of the clusters.

We also compared CMINT against two simpler base line approaches that we implemented, CLUSTER-FIRST and MERGE-FIRST. We used the three-cell type mouse reprogramming data for these comparisons. We assessed cluster quality based on Silhouette index and cluster transitions based on F-score, Precision and Recall. To obtain true transitions, we simulated data from CMINT's generative model that was learned on the reprogramming data with $k=15$ modules with a linear trajectory. This simulation enabled us to study the ability of methods to recover transitions in a controlled setting where we knew that there is a dependency between cell types.

Rule-based approach to characterize switching genes

The output of CMINT is a set of module IDs, which are matched between cell types. That is, module *i* in cell type A is associated with module *i* in cell type B. This allowed us to generate patterns denoted by the string of module assignments in each cell type. We used simple rules such as module ID=*X* in cell type iPSC and module ID=*Y* in cell type pre-iPSC and module ID=*Z* in cell type MEF, as well as more complex rules that would group together different module IDs in to the same class. For example, for the reprogramming system, module IDs <=5 were all associated with repressive marks and to find genes that transitioned from activating modules to repressive modules between cell type iPSC and cell type MEF, we used the rule "genes with module assignment <=5 in iPSC AND genes with module assignment >7 in MEF". We used similar rules to define transition points in the hematopoietic system as well. To automate

the identification of such gene sets we have provided utility scripts to cluster the CMINT module assignments and visualize the outputs as part of the Supplementary material.

Enrichment analysis

For the reprogramming study, we examined gene sets associated with each of the modules as well as with specific rules (described above) for enrichment of various curated gene sets, presence of motif instances from the MsigDB database (Liberzon et al. 2011) and ChIP-seq peaks of known pluripotency factors (Chen et al. 2008). To test for enrichment we used the Hypergeometric test with Benjamini Hochberg procedure for multiple hypothesis correction to compute a false discovery rate (FDR). We used an FDR<0.05 to call a module or gene set to be enriched in a specific curated set. We obtained ChIP-seq datasets from Chen et al (GSE11431), which examined 15 transcription factors in mouse ESC. We used the MOSAICs peak caller algorithm (Kuan et al. 2011) to call peaks on this dataset by re-aligning the data to the mm9 genome assembly using one of two options: IO1 and TS. We mapped the peaks to genes by using a +-5000 bp window around the transcription start site (TSS) of a gene.

For the hematopoietic study, we used both Gene Ontology processes (Ashburner et al. 2000) and *cis*-regulatory elements from ORegAnno (Lesurf et al. 2016) to interpret each module. To map regions to ORegAnno we required the sequence element to lie with 1 bp overlap of the genomic regions. 243,214 regions could be mapped corresponding to ~20% of the total regions. To perform gene ontology enrichment, we mapped regions to genes based on Ensemble annotations of genic coordinates. A region could be mapped to multiple genes in which can we used all the genes associated with the region. A gene could also be associated with multiple regions and therefore cluster assignments. We kept all the cluster assignments and thus a gene could belong to multiple clusters. Our Gene Ontology enrichment was not as informative as the *cis*-regulatory element analysis, which can be improved with a better mapping of

regions and genes. Therefore, we focused on ORegAnno enrichments. We used Benjamini-Hochberg procedure corrected Hypergeometric test p-values to assess enrichment in each module.

REFERENCES

Ashburner, M. et al., 2000. Gene ontology: tool for the unification of biology. The Gene Ontology Consortium. *Nature genetics*, 25(1), pp.25–29.

Chen, X. et al., 2008. Integration of external signaling pathways with the core transcriptional network in embryonic stem cells. *Cell*, 133(6), pp.1106–1117.

Ernst, J. & Kellis, M., 2010. Discovery and characterization of chromatin states for systematic annotation of the human genome. *Nature Biotechnology*, 28(8), pp.817–825.

Kuan, P.F. et al., 2011. A Statistical Framework for the Analysis of ChIP-Seq Data. *Journal of the American Statistical Association*, 106(495), pp.891–903.

Lara-Astiaso, D. et al., 2014. Chromatin state dynamics during blood formation. *Science (New York, N.Y.)*, 345(6199), pp.943–949.

Lesurf, R. et al., 2016. ORegAnno 3.0: a community-driven resource for curated regulatory annotation. *Nucleic Acids Research*, 44(Database issue), pp.D126–D132.

Liberzon, A. et al., 2011. Molecular signatures database (MSigDB) 3.0. *Bioinformatics (Oxford, England)*, 27, pp.1739–1740.

Maherali, N. et al., 2007. Directly reprogrammed fibroblasts show global epigenetic remodeling and widespread tissue contribution. *Cell Stem Cell*, 1(1), pp.55–70.

Sridharan, R. et al., 2009. Role of the Murine Reprogramming Factors in the Induction of Pluripotency. *Cell*, 136(2), pp.364–377.

Yu, P. et al., 2013. Spatiotemporal clustering of the epigenome reveals rules of dynamic gene regulation. *Genome research*, 23(2), pp.352–364.
